# Tet methylcytosine dioxygenase 2 (TET2) deficiency elicits EGFR-TKI (tyrosine kinase inhibitors) resistance in non-small cell lung cancer

**DOI:** 10.1038/s41392-024-01778-4

**Published:** 2024-03-09

**Authors:** Jian Zhang, Kejia Zhao, Wenjing Zhou, Ran Kang, Shiyou Wei, Yueli Shu, Cheng Yu, Yin Ku, Yonghong Mao, Hao Luo, Juqin Yang, Jiandong Mei, Qiang Pu, Senyi Deng, Zhengyu Zha, Gang Yuan, Shensi Shen, Yaohui Chen, Lunxu Liu

**Affiliations:** 1 Department of Thoracic Surgery and Institute of Thoracic Oncology, West China Hospital of Sichuan University, Chengdu, 610097 China; 2 Western China Collaborative Innovation Center for Early Diagnosis and Multidisciplinary Therapy of Lung Cancer, Sichuan University, Chengdu, 610097 China; 3 Biobank of West China Hospital, Sichuan University, Chengdu, 610041 China

**Keywords:** Lung cancer, Cancer therapy

## Abstract

Despite epidermal growth factor receptor (EGFR) tyrosine kinase inhibitors (TKI) have shown remarkable efficacy in patients with *EGFR*-mutant non-small cell lung cancer (NSCLC), acquired resistance inevitably develops, limiting clinical efficacy. We found that TET2 was poly-ubiquitinated by E3 ligase CUL7^FBXW11^ and degraded in EGFR-TKI resistant NSCLC cells. Genetic perturbation of TET2 rendered parental cells more tolerant to TKI treatment. TET2 was stabilized by MEK1 phosphorylation at Ser 1107, while MEK1 inactivation promoted its proteasome degradation by enhancing the recruitment of CUL7^FBXW11^. Loss of TET2 resulted in the upregulation of TNF/NF-κB signaling that confers the EGFR-TKI resistance. Genetic or pharmacological inhibition of NF-κB attenuate the TKI resistance both in vitro and in vivo. Our findings exemplified how a cell growth controlling kinase MEK1 leveraged the epigenetic homeostasis by regulating TET2, and demonstrated an alternative path of non-mutational acquired EGFR-TKI resistance modulated by TET2 deficiency. Therefore, combined strategy exploiting EGFR-TKI and inhibitors of TET2/NF-κB axis holds therapeutic potential for treating NSCLC patients who suffered from this resistance.

## Introduction

Somatic activating mutations, such as exon 21 L858R point mutation and exon19 deletion, in the epidermal growth factor receptor (EGFR) are observed in ~15% of Caucasian patients and up to 50% of East-Asian patients with NSCLC. These mutations are particularly prevalent among females, non-smokers, or former light smokers.^1^ Targeting those actionable mutations with EGFR-tyrosine kinase inhibitors (EGFR-TKIs), such as gefitinib, erlotinib, afatinib, and osimertinib, has shown impressive responses rates of 70–75% in patients with lung adenocarcinoma, with notable improvements in progression free survival ranging from 10 to 14 months when compared to standard chemotherapy.^2–5^

However, drug tolerant clones emerge through spatial and temporal clonal selection or evolve from sensitive cancer cells following initial responses to targeted therapy. The latter scenario is more common than the selection of preexisting drug resistant subclones, a phenomenon known as acquired resistance.^6,7^ Although the mechanisms of TKI acquired resistance, such as secondary mutations within the *EGFR* allele, alterations of other RTKs or downstream effectors, bypass signaling activation, and phenotypic transformation,^7^ have been well established, the acquired resistance occurs in about one third of NSCLC without any of these alterations.^8^

Accumulating evidence has documented that aberrant DNA methylation on CpG islands, m^6^A methylation on mRNA, or deregulated histone modification promotes acquired resistance.^9–11^ Su et al. conducted a study using human samples, identifying 216 CpG sites with varying DNA methylation levels to investigate their correlation with the characteristics and EGFR-TKI response status of patients with lung adenocarcinoma. Notably, methylation levels of *HOXB9* in the enhancer region demonstrated 88% sensitivity in predicting drug response. Specifically, higher methylation levels of *HOXB9* were associated with poorer responses to EGFR-TKI treatment.^12^ FTO, a N6-methyladenosine (m^6^A) eraser, was overexpressed during the development of resistant phenotypes when leukemia cells were treated by TKI therapy. In terms of mechanism, FTO-dependent removal of m6A methylation boosts the stability of mRNA molecules carrying m6A modifications associated with proliferation/survival. This, in turn, results in heightened protein synthesis.^11^ KDM5A, a histone demethylase, was also reported to be indispensable for the IGF-1R mediated emergence of drug tolerant in persister cells. Of note, Sharma et al. illustrated that chromatin regulators hold potential to dynamically modulate the drug tolerant state of cancer cells.^13^ Similarly, the decreased activity of histone methyltransferases G9a and SUV39H1 leads to the reprogramming of branched-chain amino acid (BCAA) metabolism that contributes to the acquired TKI resistance, also revealed that the development of drug resistance triggered by BCAA disorder is closely linked to alterations in H3K9 demethylation.^14^

Epi-modifications of 5-methylcytosine (5mC) and 5-hydroxymethylcytosine (5hmC) on DNA are frequently altered in cancers.^15,16^ Ten-eleven translocation proteins (TET1, TET2, TET3) are responsible for the conversion from 5mC to 5hmC, and its derivatives, 5-formylcytosine and 5-carboxylcytosine.^17–19^ It is well documented that inactivating mutations and downregulated expression or activity of TET proteins is closely associated with a wide range of cancers, including hematological malignancies and solid tumours.^16,20^ However, the function of TET proteins or their catalytic derivatives (e.g., 5hmC) in TKI resistance has not been fully addressed. In this study, we characterized the regulatory networks involving TET2 and elucidated its pivotal role in governing EGFR-TKI resistance in NSCLC. Further, we propose a therapeutic strategy that combines EGFR-TKI treatment with inhibitors targeting the TET2/NF-κB axis, offering potential for overcoming this resistance in NSCLC patients.

## Results

### Downregulation of TET2 in EGFR-TKI resistant NSCLC harboring *EGFR*-activating mutation

To define the underlying mechanism of acquired resistance to EGFR-TKI, we generated two osimertinib-resistant cell lines (PC-9OR, HCC827-OR) from parental NSCLC cell lines (PC-9, HCC827) that carry with activating *EGFR* mutation, using the dose-escalation method.^21^ We found both of the osimertinib-resistant cell lines were also cross resistant to gefitinib (Supplementary Fig. 1a–d). Whole exome sequencing (WES) revealed no significant change in their patterns of single-nucleotide variants between resistant and treatment naïve cell lines (Supplementary Fig. 1e, f). Except the intrinsic EGFR exon 19 deletion, no mutations in reported TKI-resistant gene sets (i.e., *EGFR* C797S, *HER2* amp, *Kras* mutation) was observed in both of these cell lines. The downstream EGFR signaling, such as MAPK and Akt/mTOR pathways, was not reactivated in the resistant cells (Supplementary Fig. 1g). Though the FGFR1^22^ overexpression was found in both of resistant cell lines, knockout of FGFR1 did not show any cytotoxic effect. Similar results were also found when treated these cells with FGFR1 inhibitor in combination with osimertinib (Supplementary Fig. 1h–j).

**Fig. 1:**
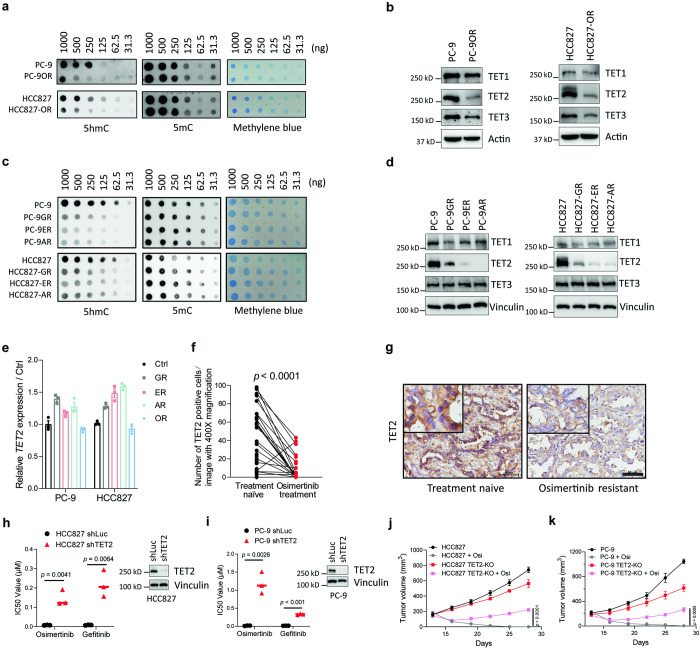
TET2 deficiency elicits EGFR-TKI resistance in NSCLC. **a** Global 5hmC levels in osimertinib-resistant (OR) and its paired treatment naïve PC-9 and HCC827 cell lines were determined by dot blot assay. The methylene blue staining was used as total genomic DNA loading control. **b** Treatment naïve and its paired OR cell lines (PC-9 and HCC827) was subjected to immunoblot (IB) analysis. **c** Global 5hmC levels in treatment naïve and its paired EGFR-TKI resistant cell lines (PC-9 and HCC827) was determined by dot blot assay. The methylene blue staining was used as total genomic DNA loading control. **d** TET2 protein expression level in indicated cell lines were determined by immunoblots. **e** mRNA level of *TET2* in indicated cells. **f** IHC score of TET2 expression in specimens from NSCLC patients who were treatment naïve or osimertinib-resistant. *n* = 30 in naïve group and *n* = 32 in resistant group. **g** Representative IHC staining of TET2 in paired specimens from one of the *EGFR-*mutant NSCLC patients as shown in (**f**). Scale bars, 50 μm. IC50 values of indicated EGFR-TKI in HCC827 (**h**) and PC-9 (**i**) cells with or without *TET2* knockdown using shRNA. Right panel, immunoblots to confirm the knockdown efficiency of *TET2* in indicated cells. Growth curves of xenograft tumors derived from HCC827 (**j**) and PC-9 (**k**) cells with or without *TET2* knockout following osimertinib treatment (2.5 mg/kg, daily, i.p.) or not (*n* = 6 mice per group). GR gefitinib-resistant, ER erlotinib-resistant, AR afatinib-resistant, OR osimertinib resistant. Statistical source data for (**f**) are shown in Supplementary Table 1. *P* values were calculated using two-tailed unpaired Student’s *t* tests (**f**, **h**, **i**) and Two way ANOVA (**j**, **k**)

Accumulating evidence demonstrates the pivotal roles of epigenetic remodeling in TKI resistance.^9–11,13,14^ We then set out to determine whether aberrant regulation of DNA, RNA, or histone modifications was occurred in resistant cells. We found the global m^6^A level of RNA in both PC-9OR and HCC827-OR cells have no difference with their parental cell lines (Supplementary Fig. 2a). While measurement of nine representative histone markers (H3K4me2 and me3, H3K9me2 and me3, H3K27me2 and me3 and ac, H3K36me2 and me3) also showed similar performance in both of paired resistant cell lines (Supplementary Fig. 2b, c). We next studied the correlation between genomic DNA methylation and the osimertinib resistance in these cell lines, and found global 5hmC but not 5mC level was dramatically decreased in osimertinib-resistant cells (Fig. 1a). Ten-eleven translocation methylcytosine dioxygenase (TET1, TET2, TET3) are responsible for the conversion of 5mC to 5hmC.^17,19^ Though TET1 and TET3 showed a tendency of decrease, TET2 was the most significantly downregulated in both resistant cell lines (Fig. 1b). The downregulation of TET2 and 5hmC were also found in PC-9 and HCC827 cells resistant to first- and second-generation of EGFR-TKIs (Fig. 1c, d and Supplementary Fig. 3). While the mRNA of *TET2* was not downregulated despite its decreased protein level in multiple EGFR-TKI resistant cell lines (Fig. 1b, d, e), indicating transcriptional-independent regulatory mechanisms. Furthermore, we found the expression of TET2 was significantly downregulated in 32 clinical specimens collected from NSCLC patients with continued disease progression after osimertinib treatment, compared with those pre-treatment NSCLC samples (Fig. 1f, g, and Supplementary Table 1), suggesting that the downregulation of TET2 was associated with the acquired resistance to EGFR-TKIs.

### TET2 deficiency elicits resistance to EGFR-TKIs in *EGFR*^*mut*^ NSCLC

To interrogate the correlation between bona fide function of TET2 and EGFR-TKI resistance, we knocked down each of *TET* genes in PC-9 and HCC827 cells by using shRNA approach. We found that downregulation of *TET2*, but not *TET1* and *TET3*, rendered these cells more tolerant to both osimertinib and gefitinib (Fig. 1h, i and Supplementary Fig. 4a–f). Using CRISPR/Cas9 system, we generated *TET2* knockout clones derived from PC-9 and HCC827 cell lines, and inoculated these cells into nude mice subcutaneously. The increased resistance to osimertinib was observed in both knockout cell lines and xenograft models (Fig. 1j, k and Supplementary Fig. 4g–i), indicating that the TET2 play a critical role in acquired resistance to EGFR-TKIs.

### TET2 was poly-ubiquitinated and degraded in EGFR-TKI resistant NSCLC cells

**Fig. 2:**
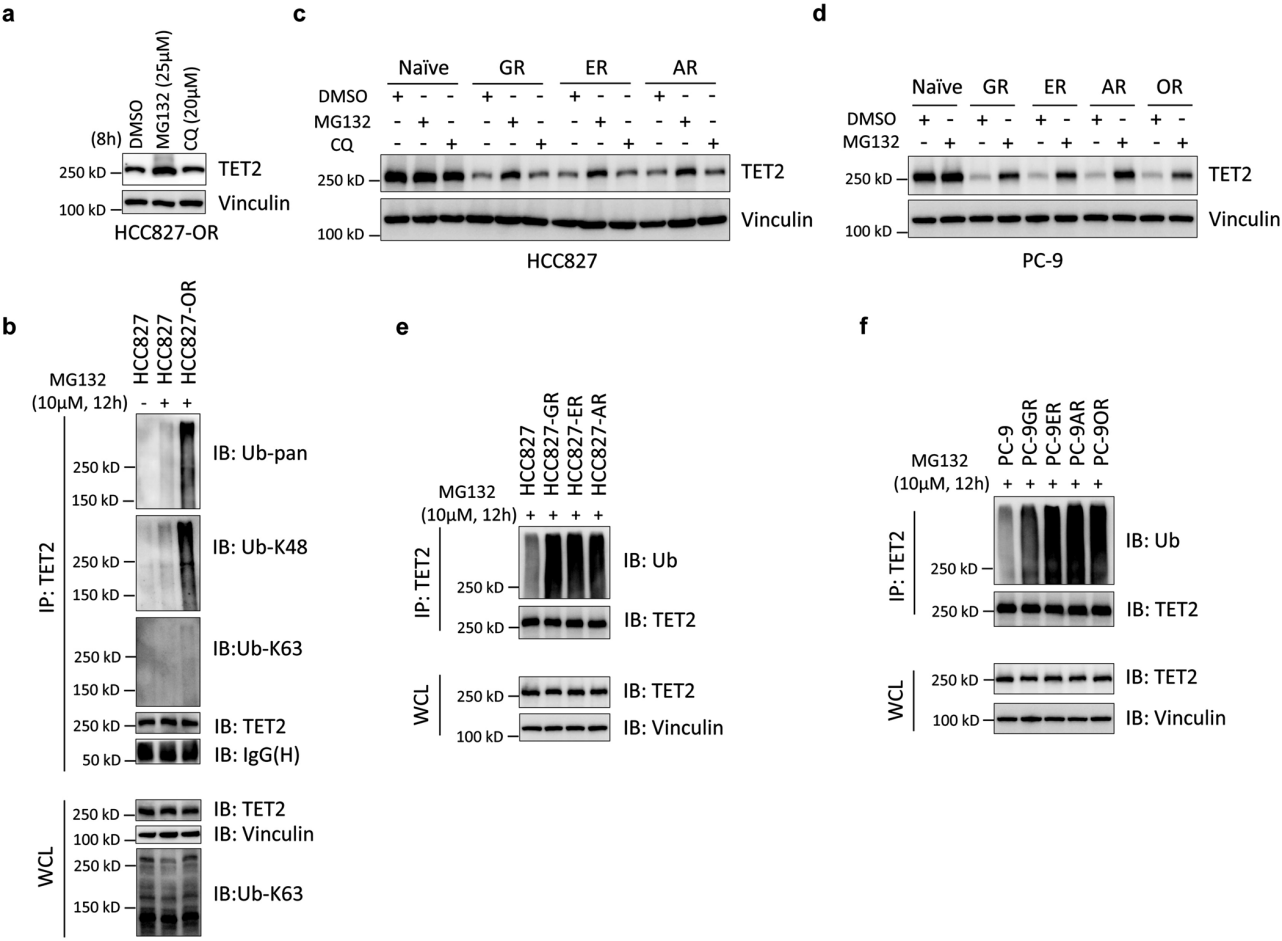
TET2 was poly-ubiquitinated and degraded in EGFR-TKI resistant NSCLC cells. **a** Immunoblot analyses of TET2 in HCC827-OR cells treated with MG132 or CQ (chloroquine) for 8 h. **b** IB analyses of TET2 IP (immunoprecipitation) products and WCL (whole cell lysate) derived from HCC827 or HCC827-OR cells treated with or without MG132 for 12 h before collection. IB analyses of TET2 in parental HCC827 (**c**) or PC-9 (**d**) cells and their corresponding EGFR-TKI resistant derivatives treated with MG132 or CQ for 8 h. IB analyses of TET2 IP products and WCL derived from parental HCC827 (**e**) or PC-9 (**f**) cells and their corresponding EGFR-TKI resistant cells treated with MG132 for 12 h before collection. All data are representative of at least two independent experiments

To characterize the mechanism of TET2 protein downregulation in EGFR-TKI resistant cells, we compared the mRNA expression of *TET2* between parental and resistant cells. No significant difference in mRNA expression was observed among these cell lines. (Fig. 1b, d, e). We found that the inhibition of proteasome activity, but not lysosome activity, substantially rescued the protein level of TET2 in osimertinib-resistant cells (Fig. 2a). The endogenous TET2-immunoprecipitation experiment showed that TET2 was highly poly-ubiquitinated which was catalyzed in a K48- but not K63-linked manner^23^ in resistant cells, compared with parental cells when treated with MG132 (Fig. 2b), indicating the downregulation of TET2 was resulted from its high level of poly-ubiquitination and, subsequently, protein degradation in osimertinib-resistant cells (Fig. 1b, d). Similar results were also found in first- and second- generation TKI resistant PC-9 or HCC827 cell lines (Fig. 2c–f), suggesting that TET2 was poly-ubiquitinated and degraded in cells resistant to multiple EGFR-TKIs.

### Inactivation of MEK1 leads to the proteasome degradation of TET2

**Fig. 3:**
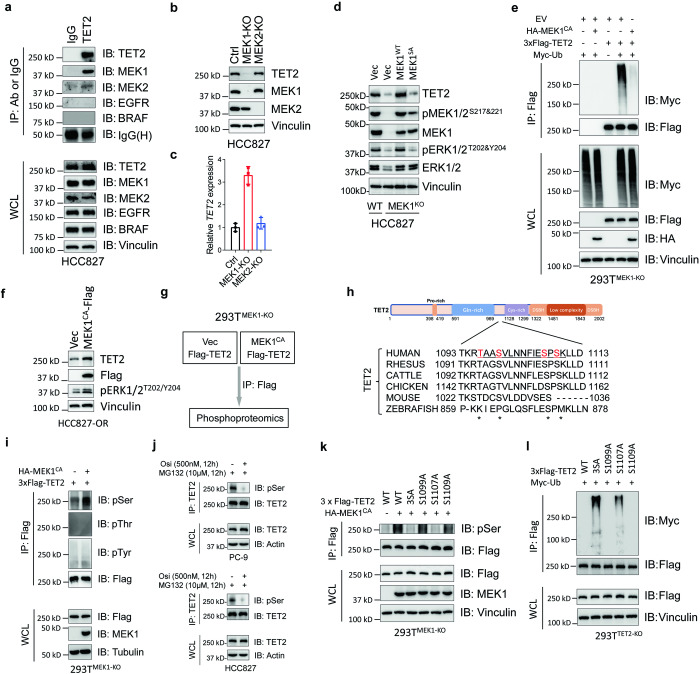
Impaired phosphorylation enhanced poly-ubiquitination of TET2. **a** IB analyses of TET2 IP products and WCL derived from HCC827 cells. IgG was used as a negative control. **b** HCC827 and its derivative cells with knockout of either *MEK1* or *MEK2* were subjected to IB analysis. **c** Relative *TET2* mRNA level in indicated cells as described in (**b**). **d** IB analyses of HCC827^WT^ cells or HCC827^MEK1-KO^ cells reintroduced with empty vector, MEK1^WT^ or MEK1^SA^. **e** CRISPR-Cas9 mediated *MEK1* knockout of 293T cells were transfected with indicated constructs and treated with MG132 (10 μM, 12 h) before IP and IB analyses. **f** IB analyses of HCC827-OR cells without or with the overexpression of constitutive MEK1 (MEK1^CA^). **g** Flow chart to show Flag IP products from 293T^MEK1-KO^ cells transfected with indicated constructs and treated with MG132 were subjected to phosphoproteomics. **h** Alignment of putative TET2 phosphorylation residues among different species. The asterisks below indicate the four potential phosphorylated amino acids. **i** IB analyses of Flag IP products from 293T^MEK1-KO^ cells treated with MG132 (10 μM, 12 h) as shown in (**g**). pSer pan phospho-serine, pThr pan phospho-threonine, pTyr pan phospho-tyrosine. **j** IB analyses of endogenous TET2 immunoprecipitated from PC-9 (up) or HCC827 (bottom) cells with or without osimertinib treatment. **k** 293T^MEK1-KO^ cells were transfected with indicated constructs and treated with MG132 (10 μM, 12 h) before harvested for Flag immunoprecipitation, the IP products and WCL were subjected to IB analyses. **l** 293T^TET2-KO^ cells were transfected with indicated constructs and treated with MG132 (10 μM, 12 h) before harvested for Flag immunoprecipitation, the IP products and WCL were subjected to IB analyses. All data are representative of at least two independent experiments

We found the EGFR pathway was suppressed in osimertinib-resistant cell lines (Supplementary Fig. 1g), indicating that the poly-ubiquitination of TET2 may be associated with the reduced activity of EGFR pathway signaling. To test this, we treated HCC827 cells with a series of inhibitors targeting EGFR or EGFR-driven downstream pathways (RAF-MEK-ERK, PI3K/Akt and JAK/STAT pathway), only small molecules targeting EGFR and BRAF showed inhibitory effect on TET2 protein level (Supplementary Fig. 5a, b). To further define the protein-protein interactions, we performed immunoprecipitation of TET2 from HCC827 cells, and found that MEK1 and MEK2 interacted with TET2 (Fig. 3a). When these two ortholog genes were knocking out individually using CRISPR/Cas9 system, we found only MEK1 regulated TET2 in a transcription-independent manner (Fig. 3b, c). Remarkably, we observed an inverse relationship between the TET2 protein level and its mRNA transcripts in *MEK1*-KO cells compared to the WT counterpart (Fig. 3b, c and Supplementary Fig. 5c–e) implying the potential involvement of downstream factors responsible for a negative feedback mechanism in transcriptional regulation. Notably, the reintroduction of MEK1^WT^ resulted in a complete restoration of TET2 levels (Fig. 3d and Supplementary Fig. 5c, d), whereas the phosphorylation-deficient form of MEK1 (MEK1^SA^, specifically MEK1^S218A&S222A^) did not yield the same effect (Fig. 3d). Furthermore, the data also revealed a positive correlation between the pMEK1^S218&S222^ level and the protein stability of TET2 (Fig. 3d), providing additional evidence for the critical role of MEK1 phosphorylation in the regulation of TET2 protein levels. In addition, the proteasome inhibitor (MG132) completely restored the TET2 protein level in HCC827^MEK1-KO^ cells, and so did the NAE inhibitor (MLN4924) even though the strength was not as strong as MG132 (Supplementary Fig. 5f). In 293T^MEK1-KO^, a cell line that *MEK1* was ablated by CRISPR/Cas9 system (Supplementary Fig. 6a), ectopic expression of constitutive active form of MEK1 (MEK1^CA^) also dramatically suppressed the poly-ubiquitination of TET2 (Fig. 3e). Likewise, restoration of MEK1 activity in HCC827-OR cells by overexpression of MEK1^CA^ partially rescued the level of TET2 (Fig. 3f). Our findings suggested the extensive regulatory axis between MEK1 and TET2, and the potential crosstalk of phosphorylation and ubiquitination in the control of TET2 protein stability.^24^

### MEK1 phosphorylates TET2 at S1107

To further characterize the specific amino acid(s) of TET2 that was phosphorylated by MEK1, we transfected the flag-tagged TET2 with or without the MEK1^CA^ into 293T^MEK1-KO^ cells. The flag complexes were immunopurified from each group of transfected cells and subjected to phosphoproteomic analyses (Fig. 3g). The TET2 peptide “[R].TAASVLNNFIESPSK.[L]” with 1xphospho [S/T] modification was only observed in MEK1^CA^ co-transfected group (Fig. 3h and Supplementary Tables 2, 3). To figure out the type (s) of phosphorylated amino acid (s), we measured the immunoprecipitated flag complexed described before with pan-serine/threonine/tyrosine antibodies and found that only pan-serine but not pan-threonine or pan-tyrosine immunoblots of flag-TET2 was enhanced in MEK1^CA^ co-transfected group (Fig. 3i), implying that only serine was phosphorylated within the indicated peptide of TET2. We also found the pan-serine signals of TET2 immunoprecipitated from EGFR^mut^ cells were diminished when treated by osimertinib (Fig. 3j), further demonstrating the association between EGFR-MEK cascade and TET2 phosphorylation. To interrogate the precise serine (s) which is (are) phosphorylated among the detected TET2 peptide, we then cloned four constructs containing S1099A, S1107A, S1109A or all three S to A (3A) mutations and found that only S1107A and 3A mutants of TET2 showed diminished signal of pan-serine immunoblots, compared with WT group in the presence of MEK1^CA^ co-transfection (Fig. 3k). Implying the S1107 in TET2 was the most like amino acid phosphorylated by MEK1. When transfected into 293T^TET2-KO^ cells, only TET2 with either S1107A or 3A mutation exhibited high level of poly-ubiquitination (Fig. 3l and Supplementary Fig. 6b). The reintroduction of constitutive phosphorylated form TET2^S1107D^ in HCC827-OR and PC-9OR cells partially restored the sensitivity to osimertinib both in vitro (both cell lines tested) and in vivo (HCC827-OR derived xenograft tested only) (Supplementary Fig. 7). Collectively, our results suggested that TET2 protein stability is enhanced by MEK1 phosphorylation at Ser^1107^.

### CUL7^FBXW11^ targets TET2 for K48-linked poly-ubiquitination mediated proteasome degradation

**Fig. 4:**
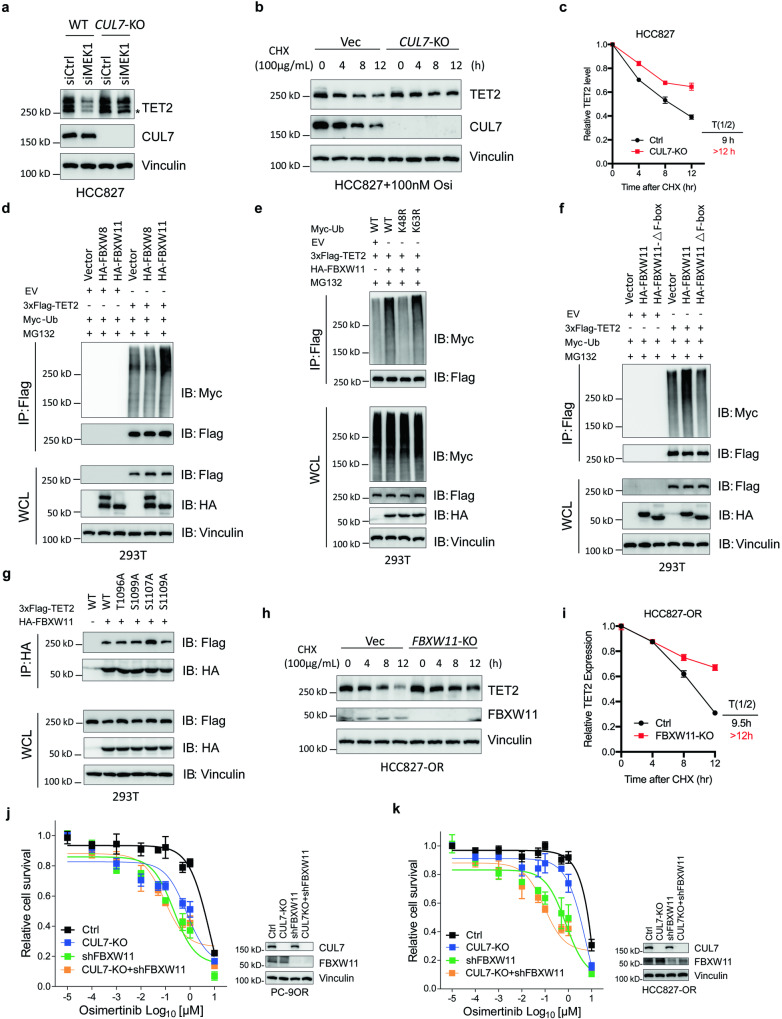
CUL7^FBXW11^ mediates K48-linked poly-ubiquitination and degradation of TET2. **a** IB analyses to determine the TET2 level in HCC827 cells without or with the knockout of *CUL7* gene treated with indicated siRNAs. The asterisk indicates the main band of TET2. **b** HCC827^WT^ or HCC827^CUL7-KO^ cells were treated with 100 μg/mL CHX (cyclohexane) and osimertinib (100 nM) prior to IB analysis. Vinculin served as a loading control. **c** TET2 protein levels were quantified by normalization to vinculin. Time of TET2 half-life (T1/2) in each group was denoted. **d**–**f** IB analyses of Flag IP products and WCL derived from 293T cells transfected with indicated constructs and treated with MG132 (10 μM, 12 h). To determine the E3 ligase substrate receptor responsible for TET2 (**d**), to measure the type of ubiquitin ligated to targeted protein (**e**), and to verify the dependence of F-box domain for the full activity of FBXW11 (**f**). **g** IB analyses of HA IP products and WCL derived from 293T cells transfected with indicated constructs and treated with MG132 (10 μM, 12 h). **h** HCC827-OR^WT^ or HCC827-OR^FBXW11-KO^ cells were treated with 100 μg/mL CHX (cyclohexane) prior to IB analysis. Vinculin served as a loading control. **i** TET2 protein levels were quantified by normalization to vinculin. Time of TET2 half-life (T1/2) in each group was denoted. All data are representative of at least two independent experiments. Relative survival curve describing the viability of PC-9OR (**j**) or HCC827-OR (**k**) cells with *CUL7* knockout or *FBXW11* knockdown or both. Immunoblots to show the knockout or knockdown efficiency of *CUL7* and *FBXW11*

To identify the E3 ligase that is responsible for the TET2 degradation, we performed mass spectrometric analyses on flag complexes immunopurified from 293T^MEK1-KO^ cells with either singularly transfected with flag-TET2, or co-transfected with flag-TET2 and MEK1^CA^ (Supplementary Fig. 8a). As expected, TET2 hits top in the list of proteins identified in mass spectrometry (Supplementary Fig. 8b). Since MLN4924, a NAE inhibitor that specifically inhibit the activity of CULLIN-based E3 ligases, rescued the protein level of TET2 caused by *MEK1* deletion (Supplementary Fig. 5f), we assess the expression of the classical scaffold proteins (CUL1, 2, 3, 4A, 4B, 5, 7) of CULLIN family in multiple TKI-resistant cell lines (PC-9OR, HCC827-OR and HCC827-GR). We found that only CUL7 was consistently upregulated in all resistant cells (Supplementary Fig. 9a–c), and CUL7 was the only protein that interacted with TET2 (Supplementary Fig. 8b). Consistently, genetic knockout of CUL7 but not the other CULLINs rescued the TET2 level in HCC827 cells when transfected with *MEK1* siRNA (Fig. 4a and Supplementary Fig. 9d–i). The half-life of TET2 was dramatically extended when *CUL7* has been depleted (Fig. 4b, c).

To identify the substrate receptor of CUL7 that was engaged in the regulation of TET2 degradation, we examined the canonical F-box containing E3 ligases of CUL7, FBXW8 and FBXW11.^25^ We found FBXW11 but not FBXW8, promoted the K48-linked poly-ubiquitination of TET2 (Fig. 4d, e), and the F-box domain of FBXW11 was indispensable for its enzymatic function (Fig. 4f). Only mutating Serine 1107 to Alanine enhanced its interaction with FBXW11 in the presence of MG132 treatment (Fig. 4g), indicating that MEK1 phosphorylates TET2 at Ser^1107^ stabilize TET2 by disrupting its interaction with FBXW11. Though no significant change in FBXW11 protein level between resistant and parental cells (Supplementary Fig. 9j), the half-life of TET2 was extended when FBXW11 was absent (Fig. 4h, i). To functionally tested the CUL7 and/or FBXW11 in drug resistance, we knocked down the *FBXW11* or knocked out the *CUL7* or did both in PC-9OR/HCC827-OR cells and found that the *FBXW11* knockdown sensitized the resistant cells to osimertinib. Knockout of *CUL7* also showed improved osimertinib sensitivity though it was inferior to *FBXW11* knockdown. However, knockout of *CUL7* did not further strengthen the drug sensitivity displayed in resistant cells with *FBXW11* knockdown (Fig. 4j, k), indicating that CUL7 and FBXW11 are functionally in the same pathway in affecting the drug sensitivity in EGFR-TKI resistant cells. These results suggested that FBXW11 is potentially an E3 ligase of TET2 that is responsible for its protein stability in NSCLC cells, and also involved in regulating the resistance to EGFR-TKIs.

### Loss of TET2 leads to the upregulation of TNF/NF-κB pathway signaling

**Fig. 5:**
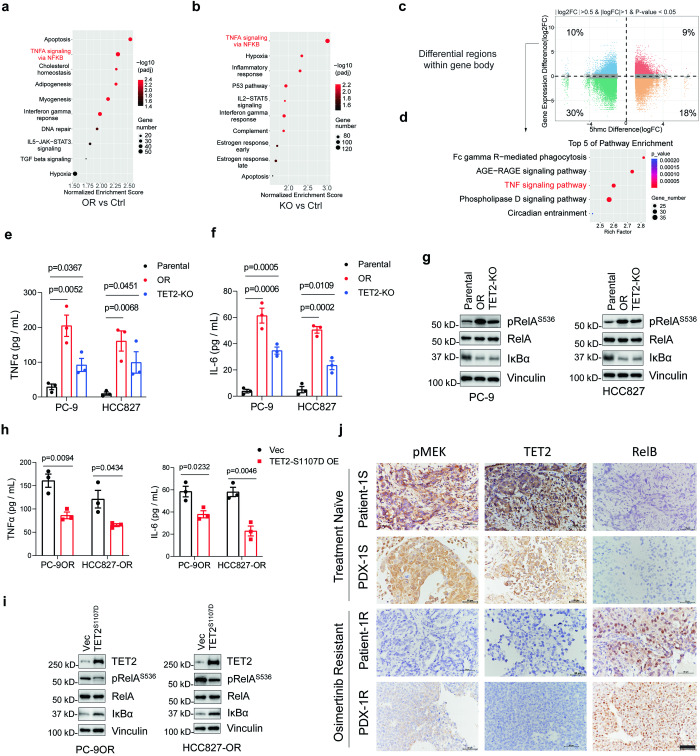
TET2 deficiency elicits the activation of TNF/NF-κB signaling. Kyoto Encyclopedia of Genes and Genomes pathway (KEGG) analyses of upregulated differential genes in HCC827-OR cells (**a**) and HCC827^TET2-KO^ (**b**) cells compared with HCC827 naïve cells. **c** Scatter plots to show the differential gene expression and the differential hypomethylated regions in gene body derived from integrated analysis of RNA-Seq and 5hmC-Seq data in HCC827 and HCC827^TET2-KO^ cells. **d** KEGG analyses of genes within differential hypomethylated regions in gene body in HCC827^TET2-KO^ cells, the gene sets analyzed are adapted from **c** shown in blue color. Enzyme-linked immunosorbent assay (ELISA) to measure the TNFα (**e**) and IL-6 (**f**) level in indicated cell lines. **g** IB analyses of pRelA^S536^, RelA and IκBα in the cell lines described in (**f**). **h** ELISA to measure TNFα and IL-6 level in PC-9OR or HCC827-OR cells with or without TET2^S1107^ overexpression. **i** IB analyses of pRelA^S536^, RelA and IκBα in the cell lines described in (**h**). **j** Representative IHC staining of pMEK, TET2, and RelB in specimens from NSCLC patients and PDXs, which were resistant (Patient-1R, PDX-1R) or sensitive (Patient-1S, PDX-1S) to osimertinib treatment. Scale bars, 50 μm. *P* values were calculated using two-tailed unpaired Student’s *t* tests (**e**, **f**, **h**). Data in (**e**–**i**) are representative of two independent experiments. Data are shown as mean ± SEM

We next sought to determine the downstream signaling of TET2 deficiency in regulating EGFR-TKI resistance. RNA sequencing (RNA-Seq) analyses were performed on parental, osimertinib-resistant and *TET2*-KO cells derived from HCC827. KEGG enrichment analysis of the significant upregulated genes from the differential analysis of *TET2*-KO versus control and osimertinib-resistant versus control, revealed TNFα signaling via NF-κB was the only significant pathway that shared between the two groups (Fig. 5a, b). The NF-κB signaling among these cell lines was further validated by quantitative real-time PCR (RT-qPCR) (Supplementary Fig. 10a). Since TET2 modulates the conversion from 5mC to 5hmC, we performed 5hmC dot blot and 5hmC sequencing (5hmC-Seq)^26^ analyses to determine the dynamic distribution of 5hmC marks between *TET2*-KO and parental cells. Downregulation of the global 5hmC level in *TET2*-KO cells was observed (Supplementary Fig. 10b, c), though the reduction of 5hmC marks was not genomic region specific (Supplementary Fig. 10d, e). Since the gene transcription activity is largely influenced by DNA methylation on either promoter or gene body,^27^ we performed an integrative analysis using both RNA-Seq and 5hmC-Seq (regions on gene body and promoter) data from *TET2*-KO and parental cells of HCC827. We found decreased 5hmC marks in a large proportion of genomic regions in *TET2*-KO cells, from the regions within gene body (decreased in 40%) to the promoter (decreased in 38%) (Fig. 5c and Supplementary Fig. 10f). Although increased 5hmC marks in *TET2*-KO cells was also observed in 27% of gene body regions and 28% of promoter regions, this upregulation might be affected by the compensatory effects of TET1 and/or TET3 (Fig. 5c and Supplementary Fig. 10f). Nevertheless, when performed gene set enrichment analysis (GSEA) of the genes with both elevated RNA expression and decreased 5hmC marks (Fig. 5c, blue), the gene sets from gene body but not promoter regions showed significant enrichment of TNF signaling pathway (Fig. 5c, d and Supplementary Fig. 10f, g), indicating TET2 regulates TNF signaling via demethylation on these genes.

Among the genes of TNF/NF-κB pathway, *TNF* and *IL-6* were the most significantly upregulated genes in EGFR-TKI resistant and *TET2*-KO cells (Supplementary Fig. 10a). The secreted protein levels of TNF and IL-6 were verified using ELISA experiments (Fig. 5e, f). Further, immunoblots also showed a tendency of NF-κB activation in resistant and KO cells, with an increase of pRelA^S536^, a marker indicating the activation of NF-κB, and a decrease of IκBα, a key factor that negatively regulate the NF-κB pathway (Fig. 5g). We also showed the re-introduction of TET2^S1107D^ in osimertinib-resistant cells could partially reverse these effects (Fig. 5h, i). However, the levels of TNF-α and IL-6 in two EGFRmut cells have not been affected with the RNA interference of *TET1* or *TET3*, further guaranteeing the cytokine changes were TET2-dependent (Supplementary Fig. 10h, i). Mechanistically, we found both TET2 chromatin immunoprecipitation quantitative PCR (ChIP-qPCR) and methylated DNA immunoprecipitation quantitative PCR (MeDIP-qPCR) revealed diminished TET2 occupancy and enhanced 5mC enrichment, respectively, and the differential regulated regions were preferentially occurred on the gene body locus of *TNF* and *IL-6* (Supplementary Fig. 11a–e), suggesting that the loss of TET2 trans-activates NF-κB pathway through impaired demethylation of *TNF* and *IL-6* within the gene body regions. Among the 32 clinical specimens from patients who suffered disease progression after the osimertinib treatment, 27 showed lower immunohistochemistry (IHC) score of TET2. Further, among the 27 specimens with decreased expression of TET2, 24 (88.9%) and 21 (77.8%) showed lower pMEK score and higher RelB score, respectively. The representative images of “TET2-high” and “TET2-low” in specimens from patients who were sensitive or resistant to osimertinib treatment were showed, respectively, with a positive correlation with pMEK and a negative correlation with RelB (Fig. 5j). Similar results were also found in the patient-derived xenograft (PDX) models (Fig. 5j).

### Targeting NF-κB attenuates the EGFR-TKI resistance driven by TET2 deficiency

**Fig. 6:**
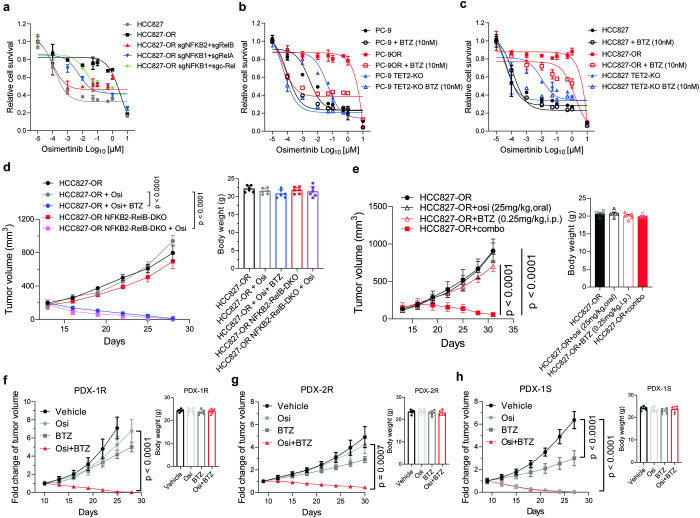
Targeting NF-κB attenuates the EGFR-TKI resistance driven by TET2 deficiency. **a**–**c** Relative survival curve describing the viability of indicated cell lines treated with the indicated concentrations of the osimertinib for 72 h. We generated different combinations of gene knockout cell lines in HCC827-OR to mimic the repression of the NF-κB pathway (**a**). Bortezomib was used to inhibit the NF-κB pathway in PC-9OR or PC9^TET2-KO^ (**b**) and HCC827-OR or HCC827^TET2-KO^ cells (**c**). **d** Growth curves of xenograft tumors derived from HCC827-OR cells with or without *NFKB2* and *RelB* double knockout following osimertinib treatment (2.5 mg/kg, daily) or in combination with bortezomib (0.25 mg/kg, daily) (*n* = 6 mice per group). **e** Growth curves of xenograft tumors derived from HCC827-OR cells treated by osimertinib (25 mg/kg, oral, daily) or bortezomib (0.25 mg/kg, daily) or combined (*n* = 5 mice per group). right panel, body weight of mice in each group at the endpoint of treatment. Growth curves of PDX-1R (**f**), PDX-2R (**g**) and PDX-1S (**h**) in NCG mice following osimertinib treatment (5 mg/kg, daily) only or bortezomib (0.25 mg/kg, daily) only or combined (*n* = 6 mice per group). The administration strategy in mice was intraperitoneal injection (i.p.) unless stated otherwise. *P* values were calculated using Two way ANOVA (**d**–**h**). Data are shown as mean ± SEM

To address the functional roles of TNF/NF-κB pathway in EGFR-TKI resistance, we knocked out the genes that activate canonical or non-canonical NF-κB pathway through dimeric complexes.^28^ Functional knockout experiments using CRISPR/Cas9 showed that the disruption of the key NF-κB factors (NFKB1, NFKB2, RelA, RelB, and c-Rel) in HCC827-OR cells significantly enhanced the sensitivity to osimertinib treatment (Fig. 6a and Supplementary Fig. 11f–h). Treatment by bortezomib, a FDA approved NF-κB inhibitor,^29^ also significantly alleviate the EGFR-TKI resistance in both osimertinib-resistant and *TET2*-KO cells (Fig. 6b, c), as well as in vivo xenograft models (Fig. 6d). We also tested the cytotoxic effect of bortezomib in combination with orally administration of osimertinib with the dose of 25 mg/kg Q.D., which was nearly equivalent to i.p. of 5 mg/kg Q.D. due to the hepatic “first pass” effect. Nevertheless, the similar results were still observed without obvious side effect because the mice weight were comparable between groups (Fig. 6e). Furthermore, we employed two osimertinib-resistant PDX (PDX-1R, PDX-2R) with no other TKI-resistant related alterations detected except the original EGFR Ex19Del and found the bortezomib combined with osimertinib treatment significantly inhibited the tumor growth (Fig. 6f, g). Interestingly, the single arm of bortezomib treatment also showed minor inhibitory effect on resistant PDX compared to the Vehicle group, and even better in the osimertinib sensitive PDX (PDX-1S) though the tumors still grew (Fig. 6f–h). These data demonstrate that targeting NF-κB can overcome the EGFR-TKI resistance triggered by TET2 deficiency.

## Discussion

TET2 is an oxygenase converting 5mC to 5hmC and its derivatives, the epi-marks governing DNA methylation status and modulating the transcription of downstream genes.^30^ In this study, we found that TET2 regulated *IL-6* and *TNF* by modulating the 5hmC level and the gene transcription activity. Accumulating evidences have demonstrated that TET2 was involved in the regulation of inflammation^31,32^ and the homeostasis of immune system^33^ through regulating *IL-6*. For example, in innate myeloid cells, TET2 selectively mediates active repression of *IL-6* during inflammation resolution by recruiting HDAC2.^31^ Also, deletion of *TET2* enhances HIV-1 replication and sustains pro-inflammatory cytokine IL-6 in myeloid cells.^34^ The transcription activity is also dependents on the modification site of epi-marks. Lian et al. showed *IL-6* expression was regulated by single CpG methylation in the downstream of transcription initiation site.^35^

A number of post-translational modifications on TET2 have been reported, including oxidative stress,^36^ diabetes,^37^ immunity,^34,38^ and cancer.^20,37^ Phosphorylation orchestrates ubiquitination is the most common mechanism of protein stability regulation which execute the upstream signal transduction.^24^ Wu et al. showed that increased glucose levels impede AMPK-mediated phosphorylation at TET2 serine 99 which leads to the destabilization of TET2.^37^ In this study, we reported that protein kinase MEK1 phosphorylated TET2 at serine 1107 preventing proteasome degradation of TET2. Our findings suggested that the phosphorylation at Ser 99 and Serine 1107 of TET2 were important for its protein stability, which is different from the conventional phosphorylation-priming for the recruitment of E3 complexes and subsequent proteasome degradation.^24^ Unlike the recent study demonstrated the YAP/TEAD was activated to counteract apoptosis when treated NSCLC in combination with EGFR and MEK inhibitors,^39^ we found the Hippo signaling has not been activated in both osimertinib-resistant cells (Supplementary Fig. 12a). Also, treated with two YAP/TEAD inhibitors XAV939^39^ or TED-347^40^ in resistant cells did not affecting the NF-κB signaling (Supplementary Fig. 12b, c), indicating that the YAP/TEAD are unlikely to modulate the activation of TNF/NF-κB signaling in our TKI-resistant cell systems. Therefore, we showed that NF-κB signaling is an alternative pathway responsible for the resistance to EGFR-TKIs through impeded activity of both EGFR and MEK.

We found only CUL7 of scaffold cullins was consistently overexpressed in three EGFR-TKI resistant cells. Knockout of CUL7 also rescued the TET2 protein level caused by MEK1 siRNA. CUL7 has been implicated in tumor initiation and progression. The overexpression of CUL7 has been reported in hepatocellular carcinoma^41^ and esophageal carcinoma.^42^ CUL7 protein level was associated with metastatic potential in breast cancer,^43^ ovarian cancer.^44^ FBXW11, one of the substrate receptors of CUL7, was also reported to promote the proliferation of lymphocytic leukemia cells and the metastasis of colorectal cancer.^45,46^ In this study, for the first time, we document the correlation between CUL7^FBXW11^ complexes and EGFR-TKI resistant phenotype (Fig. 4j, k). Although we showed CUL7^FBXW11^ is the E3 ligase of TET2 in NSCLC, other potential E3 ligase or substrate receptor of TET2 may exist in other cell types, such as immune cells.^34,38^ In our results, MLN4924 partially but not completely rescued the TET2 level caused by MEK1 knockout in NSCLC cells (Supplementary Fig. 5f), indicating other potential non-cullin-based E3 ligase of TET2 may also involve in this resistant mechanism, which warrants future study.

**Fig. 7:**
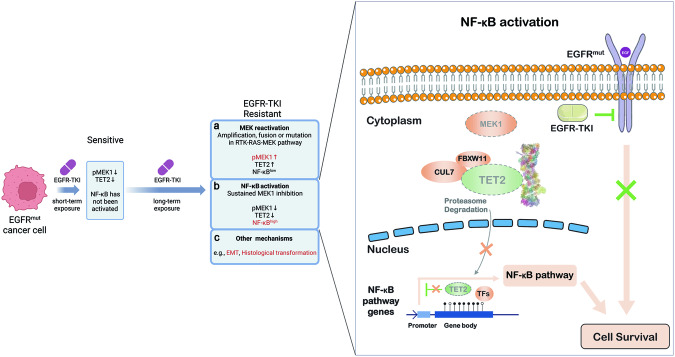
Working model. This model depicts the pMEK, TET2 and NF-κB expression patterns in *EGFR*^*mut*^ cancer cells treated by EGFR-TKI. Short-term exposed to EGFR-TKI inhibit MEK activity and downregulate TET2 whereas the NF-κB has not been evoked in *EGFR*^*mut*^ cancer cells. Nevertheless, various TKI-resistant mechanisms were reported and can be summarized in at least three aspects: (**a**) MEK reactivation, with the most cases of amplifications, fusions or mutations among RTK-RAS-MEK pathway; (**b**) NF-κB activation, featured by sustained MEK1 inhibition and the following TET2 low expression and NF-κB activation. **c** Other mechanisms, like EMT or histological transformation. The picture at the right was a further illustration of (**b**). In brief, the loss of TET2 mediated by MEK1 inactivation resulted in the upregulation of NF-κB pathway thus elicits resistance to EGFR-TKIs. This figure includes a portion generated from BioRender (https://app.biorender.com/)

Acquired resistance to EGFR-TKIs can be derived from the genetic alterations in EGFR itself, EGFR downstream genes, bypass RTK genes or cell cycle genes, which accounts for ~45% of cases, and oncogenic targets of these alterations has been exploited to develop next-generation drugs^47^ or to employ the combination therapy.^48,49^ The histologic transformation (e.g., SCLC) resistant phenotypes arise in ~15% of cases that may be treated by combined chemotherapy.^49,50^ However, the resistance mechanism for the rest of ~40% of cases is still unclear, which largely limits the potential for developing effective therapeutics due to the lack of molecular targets.^8^ Here, we showed a novel EGFR-TKI resistant mechanism with chronic activation of NF-κB signaling elicited by TET2 deficiency. And our model exemplifies that the cell growth associated protein kinase MEK1 stabilizes the pivotal epigenetic factor TET2, and the TET2/NF-κB axis represents an alternative path to confer the non-mutational acquired EGFR-TKI resistance (Fig. 7).

## Materials and methods

### Cell culture studies

The PC-9, HCC827, and HEK293T cell lines were procured from the Shanghai Academy of Science (Shanghai, China). PC-9 and HCC827 cells were cultured in Roswell Park Memorial Institute Medium–1640 (Gibco, United States), while HEK293T cells were cultured in Dulbecco’s Modified Eagle Medium (Gibco, United States). The complete medium consisted of 8–10% fetal bovine serum (FBS; Gibco, United States) and 1% penicillin-streptomycin (Gibco, United States). The cell lines were maintained at 37 °C with 5% carbon dioxide.

### Animal studies

The mice were accommodated in facilities authorized by the Animal Care and Use Committee of West China Hospital, Sichuan University. Ethical clearance for all in vivo animal experiments was authorized by the Animal Ethics Committee of West China Hospital, Sichuan University (Approval No. 2021050A), adhering to institutional protocols for animal welfare. Six-week-old male nude mice were procured from GemPharmatech Co., Ltd. (Jiangsu, China) and utilized for the study. These mice were subcutaneously inoculated with 5 × 10^6 cells until visible tumors developed.

For the HCC827^TET2-KO^ and PC-9OR^TET2-KO^ xenograft study, when the tumor volume reached 200 mm^3^ in size, mice (*n* = 6 per group) were treated with vehicle or osimertinib (2.5 mg/kg body weight, daily) via intraperitoneal injection for 27 days.

For HC827-OR^NFKB2-RelB-KO^ xenograft study, when the tumor volume reached 200 mm^3^ in size, mice (*n* = 6 per group) were treated with vehicle or osimertinib (2.5 mg/kg body weight, daily) or osimertinib in combination with bortezomib (0.25 mg/kg, daily) via intraperitoneal injection for 27 days.

The PDX model (PDX-1R and PDX-2R, carried with *EGFR*^mut^ allele and osimertinib resistant, PDX-1S, carried with *EGFR*^*mut*^ allele and osimertinib sensitive) was established in our lab according to a previous publication (Wang, D. et al., Int J Cancer. 2017). Fragments of PDX tumors were sectioned into pieces measuring 2 mm in diameter and then implanted subcutaneously into the right flanks of 6-week-old male nude mice sourced from GemPharmatech. Once the tumors reached a volume of 200–300 mm^3^, mice were randomly assigned to receive treatment in four to five different arms. Treatment options included vehicle, osimertinib (administered at 2.5 mg/kg body weight, daily), or a combination of osimertinib and bortezomib (administered at 0.25 mg/kg, daily) via intraperitoneal injection for a duration of 21 days.

Tumor volume was calculated using the following formula: *V* (mm^3^) = (*π*/6) * *L* * *W*^2^, where *L* and *W* referred to the longest longitudinal and transverse diameters, respectively.

### Human samples

This study has been ethically authorized by the institutional review committees of West China Hospital, Sichuan University. Written informed consent was obtained from all patients participating in the study. A total of 41 biopsy specimens were collected, consisting of 30 samples obtained prior to EGFR TKI treatment to establish baseline data, and 32 samples obtained from patients who developed acquired EGFR TKI resistance. Among these, 21 samples were matched samples obtained from individual patients. More detailed clinical information of lung tumors in patients is listed in Supplementary Table 1. Immunostaining of TET2 was blindly scored.

The human lung tumors used for PDX establishment were from West China Hospital, Sichuan University. The tumor collection and transplantation were authorized by Institutional Review Boards (No. 20221789) and patients gave written informed consents. The PDX-1R (ADC, EGFR Exon19Del, with distant metastasis to brain, liver and bone) was derived from a 62-year-old male and primarily resistant to osimertinib. PDX-2R (ADC, EGFR Exon19Del) was derived from a patient progressed from chemotherapy and osimertinib treatment. PDX-1S (ADC, EGFR Exon19Del) was derived from a 65-year-old female who has never been treated by chemotherapy or EGFR-TKIs. The WES data of PDX have been deposited with links to BioProject accession number PRJNA1043048 in the NCBI BioProject database. The gene alterations of the three PDXs were listed in Supplementary Table 4, note that the alterations in PDX-1R and PDX-2R were numerous due to the lack of gene mutation spectrum of paired normal tissue or blood to eliminate the redundant information of gene alterations. Even so, there were no TKI-related gene alterations existed.

### Antibodies

The following primary antibodies were used: 5mC (Eurogentec, BI-MECY-0100), 5hmC (Active Motif, 39092), m^6^A (HuaBio, HA721152), TET1 (GeneTex, GTX627420), TET2 (CST, #18950), TET3 (GeneTex, GTX121453), H3 (CST, #9715), H3K4me2 (Abcam, ab32356), H3K4me3 (Abcam, ab8580), H3K9me2 (Abcam, ab1220), H3K9me3 (Abcam, ab8898), H3K27me2 (Abcam, ab24684), H3K27me3 (Abcam, ab6002), H3K27ac (Abcam, ab4729), H3K36me2 (Abcam, ab9049), H3K36me3 (Abcam, ab9050), Ub-Pan (CST, #3936), Ub-K48 (CST, #4289), Ub-K63 (CST, #5621), Flag (CST, #14793), HA (CST, #3724), Myc (CST, #2278), Anti-Phosphoserine (Sigma, #05-1000), Anti-Phosphothreonine (Sigma, P3555), Anti-Phosphotyrosine (CST, #9411), CUL1 (HuaBio, ET1705-82), CUL2 (HuaBio, ET7108-47), CUL3 (Abclonal, A19623), CUL4A (CST, #2699), CUL4B (Abclonal, A12696), CUL5 (Abclonal, A5369), CUL7 (SantaCruz, sc-53810), FBXW11 (Proteintech, 13149-1-AP), pEGFR (#2234, CST), EGFR (CST, #4267), pMEK (CST, #9154), MEK1 (CST, #2352), MEK2 (CST, #9147), BRAF (CST, #9433), pAKT (CST, #4060), AKT (CST, #9272), pERK (CST, #4376), ERK (CST, #9102), p-mTOR (SantaCruz, sc-517464), mTOR (SantaCruz, sc-517464), pS6K (CST, #9234), S6K (CST, #2078), pFGFR (CST, #3471), FGFR1 (CST, #9740), pPDGFRβ (CST, #3124), PDGFRβ (CST, #3169), pMET (CST, #4033), MET (CST, #8041), pALK (CST, #3341), ALK (CST, #3791), pROS1 (CST, #3078), ROS1 (CST, #3266), pAMPK (CST, #2523), pS6 (CST, #2215), pRb (CST, #8516), pSTAT1 (CST, #7649), pSTAT3 (CST, #9145), NFKB-p50/p105 (HuaBio, ET1603-18), NFKB2-p52/p100 (CST, #37359), pRelA (CST, #3033), RelA (HuaBio, ET1603-12), RelB (HuaBio, ET1612-18), c-Rel (HuaBio, ET1705-44), IκBα (CST, #4812), Vinculin (CST, #13901), β-Actin (ZSGB-BIO, TA-09), β-Tubulin (ZSGB-BIO, TA-10).

### Reagents

Osimertinib (S7297), gefitinib (S1025), erlotinib (S7786), afatinib (S1011), BGJ398 (S2183), bortezomib (S1013), Cycloheximide (S7418), MG132 (S2619), chloroquine (S6999) and MLN4924 (S7109) were purchased from Selleckchem. Crizotinib (HY-50878), LY3009120 (HY-12558), PD0325901 (HY-10254), SCH772984 (HY-50846), Wortmannin (HY-10197), MK-2206 (HY-108232), Rapamycin (HY-10219), Ruxolitinib (HY-50856), XAV-939 (HY-15147) and TED-347 (HY-125269) were from MedChemExpress.

### Plasmids and siRNAs

pRK7-3xFlag-TET2 (WT, T1096A, S1099A, S1107A, S1107D, S1109A and S1099A/S1107A/S1109A (3A) mutants), pRK7-HA-MEK1^CA^, pCDNA3.1-Myc-Ub (WT, K48R, and K63R mutants), pRK7-HA-FBXW8, pRK7-HA-FBXW11 (WT and F-Box-deletion mutant), pCDH-MEK1^CA^-Flag, pCDH-MEK1^SA^-Flag, and pCDH-3xFlag-TET2 (WT and S1107D mutant) were generated in this study.

The oligos used for short-hairpin RNAs were annealed and cloned into the pLKO.1 backbone. The primers were listed in the following table.

**Table:** 

Gene	Forward primer (5′->3′)	Reverse primer (5′->3′)
Luciferase	CACTTACGCTGAGTACTTCGA	TCGAAGTACTCAGCGTAAGTG
hTET1	CCTTGATAGAATCACTCAGTT	AACTGAGTGATTCTATCAAGG
hTET2	CCTTATAGTCAGACCATGAAA	TTTCATGGTCTGACTATAAGG
hTET3	GGTCGTATAATGGCATATTAA	TTAATATGCCATTATACGACC
hFBXW11	TCGTACTCTCAATGGGCACAA	TTGTGCCCATTGAGAGTACGA

The oligos used for guide RNAs were annealed and cloned into the LentiCRISPR v2 backbone. The primers were listed in the following table.

**Table:** 

Gene	Forward primer (5′->3′)	Reverse primer (5′->3′)
hTET2	AAGTAAATGGAGACACCAAG	CTTGGTGTCTCCATTTACTT
hMEK1	TATGGTGCGTTCTACAGCGA	TCGCTGTAGAACGCACCATA
hMEK2	GATCTCCCCGTCACTGTAGA	TCTACAGTGACGGGGAGATC
hCUL1	GAACTCCAGCTCCTCGTGCT	AGCACGAGGAGCTGGAGTTC
hCUL2	TTTGACGACAATAAAAGCCG	CGGCTTTTATTGTCGTCAAA
hCUL3	ATGTCAGTTCACGTCAAAAC	GTTTTGACGTGAACTGACAT
hCUL4A	CCTCTAAGCGTTTACTTACA	TGTAAGTAAACGCTTAGAGG
hCUL4B	ATTCAATGCTACCCTCCATT	AATGGAGGGTAGCATTGAAT
hCUL5	CCCTCGTATTTACAACAAAA	TTTTGTTGTAAATACGAGGG
hCUL7	TGAACACCCCATGTCTTTCG	CGAAAGACATGGGGTGTTCA
hNFKB1	CCATCCCATGGTGGACTACC	GGTAGTCCACCATGGGATGG
hNFKB2	TAGGCTGTTCCACGATCACC	GGTGATCGTGGAACAGCCTA
hRelA	AGCGCCCCTCGCACTTGTAG	CTACAAGTGCGAGGGGCGCT
hRelB	CCCGCGTGCATGCTTCGGTC	GACCGAAGCATGCACGCGGG
h-c-Rel	CCTCATCCTCATGATTTAGT	ACTAAATCATGAGGATGAGG
hFGFR1	GATCCGGTCAAATAATGCCT	AGGCATTATTTGACCGGATC
hFBXW11	CATCACTTTACGCCGTGTCC	GGACACGGCGTAAAGTGATG

For siRNAs: siCtrl (5′-GUCGAGCAGUACCUAUUCU-3′) and siMEK1 (5′-GCAACUCAUGGUUCAUGCU-3′) were synthesized in Tsingke Biological Technology.

### Immunoblotting

Fresh-frozen tissue or cell samples were lysed using RIPA buffer (Beyotime, Shanghai, China) supplemented with protease and phosphatase inhibitor cocktails (Beyotime, Shanghai, China). The protein concentration was measured using a bicinchoninic acid (BCA) protein assay kit (Beyotime, Shanghai, China). Equal amounts of cell lysate (15–30 µg) were loaded onto appropriate concentration of polyacrylamide gels containing 0.1% sodium dodecyl sulfate (SDS) and subsequently transferred to polyvinylidene fluoride membranes or nitrocellulose membranes (Merck Millipore, Cork, IRL). The membranes were then blocked with 1X phosphate buffer saline (PBS) containing 0.1% Tween-20 and 5% skim milk at room temperature for about 1 h and incubated with indicated antibodies diluted by 1X PBS or commercial antibody diluent (Beyotime, Shanghai, China) overnight at 4 °C.

### Dot blot

For DNA blotting. Denature of genomic DNA at 100 °C for 5 min and move on ice immediately. Load 2 µL DNA on nitrocellulose membrane and air dry. Then following the standard immunoblotting procedures. The dilution rate is 1:1000 for 5mC and 5hmC antibody.

For RNA blotting. Denature of total RNA at 100 °C for 5 min and move on ice immediately. Load 2 µL RNA on nitrocellulose membrane. Crosslink RNA on membrane with a UV light (15 W) for 5 min. Then following the standard immunoblotting procedures. The dilution rate is 1:1000 for m^6^A antibody.

### Immunohistochemistry (IHC)

Tumor sections were subjected to deparaffinization, rehydration, and treatment with 0.3% hydrogen peroxide methanol solution for 20 min to inhibit endogenous peroxidase activity. Subsequently, sections were blocked with goat serum and incubated overnight with primary antibodies against pMEK (CST, #9154, 1:200), TET2 (CST, #18950, 1:100), or RelB (HuaBio, ET1612-18, 1:100). After washing, sections were incubated with horseradish peroxidase-conjugated anti-rabbit antibody (ZSGB-BIO, Beijing, China), followed by staining with diaminobenzidine solution (ZSGB-BIO) to visualize the horseradish peroxidase (HRP) activity. Counterstaining with hematoxylin was performed before mounting. Negative controls were prepared by incubating samples with PBS instead of primary antibodies. Immunostained sections were evaluated independently by at least two researchers in a blinded manner. The intensity and frequency of staining were integrated, and the number of positively stained cells in a representative image at 400× magnification was used to determine the IHC score.

### Cell viability assay

Cells were seeded in triplicate in 96-well plate the day before and then given different doses of indicated compounds and cultured for another 3–5 days. Cells were then stained with Cell Counting Kit-8 (CCK-8) reagent and assessed with Epoch2 multi-volume spectrophotometer system (450 nm) after 1 h incubation. The growth ratio was determined by normalizing the fluorescence signal obtained after subtracting the fluorescence signal of the blank.

### Immunoprecipitation

Cells were lysed using EBC buffer (composed of 50 mM Tris pH 7.5, 120 mM NaCl, 0.5% NP-40), supplemented with a protease inhibitor cocktail (Solarbio, P6730), and phosphatase inhibitors (Solarbio, P1260). The protein concentration was determined using a BCA protein assay (BCA) kit (Solarbio, Beijing, China). For immunoprecipitation analysis, 1000 µg of total cell lysates were incubated with primary antibodies overnight. Then protein A/G beads were added for another 6 h incubation in a rotator at 4 °C. The immunocomplexes recovered were washed four times with NETN buffer (20 mM Tris, pH 8.0, 100 mM NaCl, 1 mM EDTA, and 0.5% NP-40) before being separated by SDS-PAGE and subjected to immunoblotting with specific antibodies.

### ELISA

In brief, for every 1 × 10^6 cells, add about 200 μL of pre-cooled PBS and fully lyse the cells by ultrasonication. Centrifuge the lysate for 10 min at 1500 × *g* at 4 °C, and then remove the cell debris to collect the supernatant. The cell culture supernatant is further centrifuged for 20 min at 1000 × *g* at 4 °C to collect the clarified supernatant. Both the collected cell lysates and supernatants are quantified using TNF (E-EL-H0109c, Elabscience) or IL-6 (E-EL-H6156, Elabscience) ELISA kits.

### RT-PCR and quantitative real-time PCR

The total RNA was extracted using Trizol reagent (Invitrogen) and then reverse transcribed into first-strand cDNA using PrimeScript™ RT reagent Kit with gDNA Eraser Kit (Takara). The synthesized cDNAs were subsequently subjected to quantitative real-time PCR (qRT-PCR) using gene-specific primers on the Real-Time PCR System (Bio-Rad) with TB Green II (Takara). Beta-actin (human) was utilized as an internal control for normalization. The primer sequences employed for qRT-PCR are provided in the accompanying table.

**Table:** 

Gene	Forward primer (5′->3′)	Reverse primer (5′->3′)
hTET1	GCGACCCTTGGTGCTAAACC	CAGGGCCTCACCATGAACTG
hTET2	ACAGAAGCAGCCACCACAGC	TCATAGGGCTGGTGCTTCCA
hTET3	CCATTCAGGACCCCGAGAAC	CCACTGAGGGTGGGTGTGAG
hNFKB1	AACAGAGAGGATTTCGTTTCCG	TTTGACCTGAGGGTAAGACTTCT
hNFKB2	ATGGAGAGTTGCTACAACCCA	CTGTTCCACGATCACCAGGTA
hRelA	ATGTGGAGATCATTGAGCAGC	CCTGGTCCTGTGTAGCCATT
hRelB	CAGCCTCGTGGGGAAAGAC	GCCCAGGTTGTTAAAACTGTGC
hTNF	CCTCTCTCTAATCAGCCCTCTG	GAGGACCTGGGAGTAGATGAG
hIL1B	ATGATGGCTTATTACAGTGGCAA	GTCGGAGATTCGTAGCTGGA
hIL6	ACTCACCTCTTCAGAACGAATTG	CCATCTTTGGAAGGTTCAGGTTG
hLTB	GGAGACGACGAAGGAACAGG	GTAGAGGTAATAGAGGCCGTCC
hCSF1	TGGCGAGCAGGAGTATCAC	AGGTCTCCATCTGACTGTCAAT
hCSF2	TCCTGAACCTGAGTAGAGACAC	TGCTGCTTGTAGTGGCTGG
hJUN	TCCAAGTGCCGAAAAAGGAAG	CGAGTTCTGAGCTTTCAAGGT
hLTBR	CTCAGCTAAATGTAGCCGCAT	ATGGTCAGGTAGTTCCAGTGC
hMAP3K14	CGGAAAGTGGGAGATCCTGAA	GGGCGATGATAGAGATGGCAG
hTRAF1	TCCTGTGGAAGATCACCAATGT	GCAGGCACAACTTGTAGCC
hTRAF3	CAGACTAACCCGCCGCTAAAG	GATGCTCTCTTGACACGCTGT
TNFRSF11A	AGATCGCTCCTCCATGTACCA	GCCTTGCCTGTATCACAAACTTT
TNFAIP3	TCCTCAGGCTTTGTATTTGAGC	TGTGTATCGGTGCATGGTTTTA
CTGF	CAGCATGGACGTTCGTCTG	AACCACGGTTTGGTCCTTGG
CYR61	CTCGCCTTAGTCGTCACCC	CGCCGAAGTTGCATTCCAG
ACTB	CCAACCGCGAGAAGATGA	CCAGAGGCGTACAGGGATAG

### Chromatin immunoprecipitation assay and data analysis

ChIP assay was performed according to the product manual of SimpleChIP® Plus Enzymatic Chromatin IP Kit (Magnetic Beads, CST, #9005). In brief, Cells were cross-linked with 1.5% formaldehyde for 20 min at room temperature, stop cross-linking by adding glycine solution. Ultrasonication to generate DNA fragments to the size of 150–900 bp. Approximately 5–10 µg of digested, cross-linked chromatin of the cell lysate were used, antibody against 5mC (Eurogentec, BI-MECY-0100), 5hmC (Active Motif, 39092), TET2 (CST, #18950), rabbit IgG (CST, #3900) or mouse IgG (CST, #5415) was added to cell lysate and incubated in a rotator at 4 °C overnight. DNA cross-linked with antibodies was then pulled down with Protein A/G beads, washed, and purified with PCR purification kit. The eluted DNA was subjected to next-generation sequencing or real-time PCR analyses.

For interaction analysis with RNA-Seq, peak located in the promoter of 5hmC is extracted, and those significant peaks are highlighted according to the intersection of genes. The screening condition is gene expression difference |log2fc| > 0.5 and 5hmC-seq difference |logfc| > 1 and *P* value < 0.05.

For quantitive PCR, primers used for ChIP-qPCR were listed in the following table.

**Table:** 

Gene	Forward primer (5′->3′)	Reverse primer (5′->3′)
hTNF-P1	ACGCTCCCTCAGCAAGGACA	GTCAGTATGTGAGAGGAAGA
hTNF-P2	GGGTGACTCCCTCGATGTTA	AACCCAAACCCAGAATTAGG
hTNF-P3	TCTCAGCTTTTTCTTTTCTC	CCCCAAATCCTAGCCCTCCA
hTNF-P4	GACCCCAGAGGGGGCTGAGG	GCCCAGACTCGGCAAAGTCG
hIL6-P1	GAGTGTCTACGTTGCTTAAG	AGGTCATCCATTCTTCACCG
hIL6-P2	ACTCTTTGTCAAGACATGCC	GACGTCCTTTAGCATGGCAA
hIL6-P3	TCCTGGTGTTGCCTGCTGCC	AGATGCCGTCGAGGATGTAC
hIL6-P4	TAGGTGATAACAATTCTGGT	ATCCAGATTGGAAGCATCCA
hIL6-P5	ATCATCCCATAGCCCAGAGC	GCTGGCATTTGTGGTTGGGT

### Protein half-life assays

Cells were subjected to transfection or specific treatments as specified. For half-life experiments, cycloheximide (100 µg/mL) was introduced into the culture medium. At designated time intervals following treatment initiation, cells were harvested, and protein levels were assessed using immunoblot analysis.

### Lentiviral production and infection

Lentivirus production involved co-transfection of lentiviral backbone constructs (8 μg) with packaging plasmid psPAX2 (6 μg) and envelope plasmid pMD2.G (4 μg) into HEK293T cells using either Lipofectamine 2000 or Polyethyleneimine. The helper plasmids were kindly provided by Prof. Ji at SIBCB, CAS. After 48 h of transfection, lentivirus-containing supernatants were collected. To remove cellular debris, the supernatants were either filtered through a 0.45 μm filtration system or centrifuged at 1500 × *g* to obtain clarified supernatant. Target cells were exposed to lentiviral supernatants supplemented with 8 μg/mL polybrene for 24 h, followed by washing. Lentivirus was added once again to enhance infection efficiency for another 24 h. Subsequently, fresh medium was added until the cells completely covered the culture dish. Cells were then selected using the appropriate antibiotic concentration.

### RNA-sequencing and data analysis

Total RNA was extracted using the RNeasy Mini Kit (Qiagen). Paired-end libraries were prepared with the TruSeq® RNA Sample Preparation Kit (Illumina, USA) according to the manufacturer’s instructions. Briefly, mRNA was purified using poly-T oligo-attached magnetic beads, fragmented, and converted into cDNA. The cDNA fragments were then end-repaired, adapters were added, and the libraries were enriched by PCR. Library quality was assessed using Qubit® 2.0 Fluorometer and Agilent 2100 bioanalyzer. Sequencing was performed on the Illumina HiSeq Xten platform. Sequencing reads were preprocessed to filter out rRNA reads, adapters, and low-quality reads. The cleaned reads were mapped to the human hg38 reference genome using Hisat2, and FPKM values for known gene models were calculated using Stringtie. Differentially expressed genes were identified using edgeR with significance determined by FDR and fold-change criteria. GSEA was performed to assess enrichment of predefined gene sets, utilizing GO, KEGG, Reactome, DO, and DisGeNET datasets. GSEA allows detection of subtle expression changes and was conducted using the GSEA analysis tool.

### Whole exome sequencing (WES) and data analysis

Genomic DNA was extracted using the QIAamp DNA Mini kit, and its quality was assessed through agarose gel electrophoresis and Qubit® DNA Assay Kit. Exome sequencing was performed using the Agilent SureSelect Human All Exon V6 kit, starting with DNA fragmentation, end repair, A-tailing, adapter ligation, and PCR amplification. The resulting libraries were hybridized with biotin-labeled probes, captured using streptomycin-coated magnetic beads, and enriched by PCR. After purification, library concentration and size distribution were measured, and libraries were sequenced on an Illumina platform.

For single-nucleotide variant allelic frequency analysis, SNPs were identified using GATK tools Mutect2 and FilterMutectCalls, with comparison to a normal female DNA sample. Non-synonymous SNPs were annotated using Oncotator, and nucleotide frequencies were extracted using bam-readcount from the filtered mutect2 output. Tumor allele frequency was estimated by dividing the number of reads encoding the tumor allele by the total number of reads at each locus, with a minimum coverage threshold of 50× for HCI002.

### LC-MS/MS analysis

LC-MS/MS analysis was conducted on a Q Exactive mass spectrometer coupled to Easy nLC for 60 min. The instrument operated in positive ion mode, acquiring MS data using a data-dependent top20 method for precursor ion selection and HCD fragmentation. Survey scans were performed at a resolution of 70,000, with HCD spectra at 17,500 resolution. MS/MS spectra were searched using the MASCOT engine against a nonredundant International Protein Index Arabidopsis sequence database. Parameters included a peptide mass tolerance of 20 ppm, MS/MS tolerance of 0.1 Da, and trypsin digestion with up to two missed cleavages. Fixed modification was Carbamidomethyl (C), and variable modification was Oxidation (M).

### Statistical analysis

The data presented are represented as means along with the standard error of the mean. To compare groups, a two way ANOVA test was conducted, as indicated in the figure legends. Correlative analyses were performed using the Spearman correlation coefficient to determine statistical significance. A significance level of *P* ≤ 0.05 was considered statistically significant. All data analyses were carried out using GraphPad Prism software (Version 9.0, GraphPad).

### Supplementary information

Sup Figures and Methods

Part of unprocessed WB images

Supplementary Table 1. Detailed clinicopathologic information of the study cohort

Supplementary Table 2. Peptides identified in TET2 transfected only group

Supplementary Table 3. Peptides identified in TET2 X MEK1 co-transfected group

Supplementary Table 4. Gene alterations of PDX obtained from WES data

## Data Availability

RNA-seq, WES, and 5hmC-seq data used in this study have been deposited in the Gene Expression Omnibus under accession code GSE223009. The WES data of PDX have been deposited with links to BioProject accession number PRJNA1043048 in the NCBI BioProject database. A portion of the raw blots and the statistical source data are included in the manuscript. However, additional data supporting the conclusions of the study can be obtained from the corresponding author upon reasonable request. The source data accompanying this paper are also provided for reference.
