# Supplementary material for: Tet methylcytosine dioxygenase 2 (TET2) deficiency elicits EGFR-TKI (tyrosine kinase inhibitors) resistance in non-small cell lung cancer
Source: Signal Transduct Target Ther. 2024 Mar 9;9:65. doi: 10.1038/s41392-024-01778-4 (PMC10924974; doi:10.1038/s41392-024-01778-4)
Supplement: Supplementary file 1 — Sup Figures and Methods [file 41392_2024_1778_MOESM1_ESM.docx]

Supplementary Materials for

**TET2 Deficiency Elicits EGFR-TKI Resistance in Non-Small Cell Lung Cancer**

Jian Zhang^1,2^, Kejia Zhao^1,2^, Wenjing Zhou^1,2^, Ran Kang^1,2^, Shiyou Wei^1,2^, Yueli Shu^1,2^, Cheng Yu^1,2^, Yin Ku^1,2^, Yonghong Mao^1,2^, Hao Luo^1,2^, Juqin Yang^3^, Jiandong Mei^1^, Qiang Pu^1^, Senyi Deng^1,2^, Zhengyu Zha^1,2^, Gang Yuan^1,2^, Shensi Shen^1,2^, Yaohui Chen^1,2,*^, Lunxu Liu^1,2,*^

Correspondence to: lunxu_liu@aliyun.com, yhchen@scu.edu.cn

**This PDF file includes:**

Materials and Methods

Supplementary Figures. S1 to S12


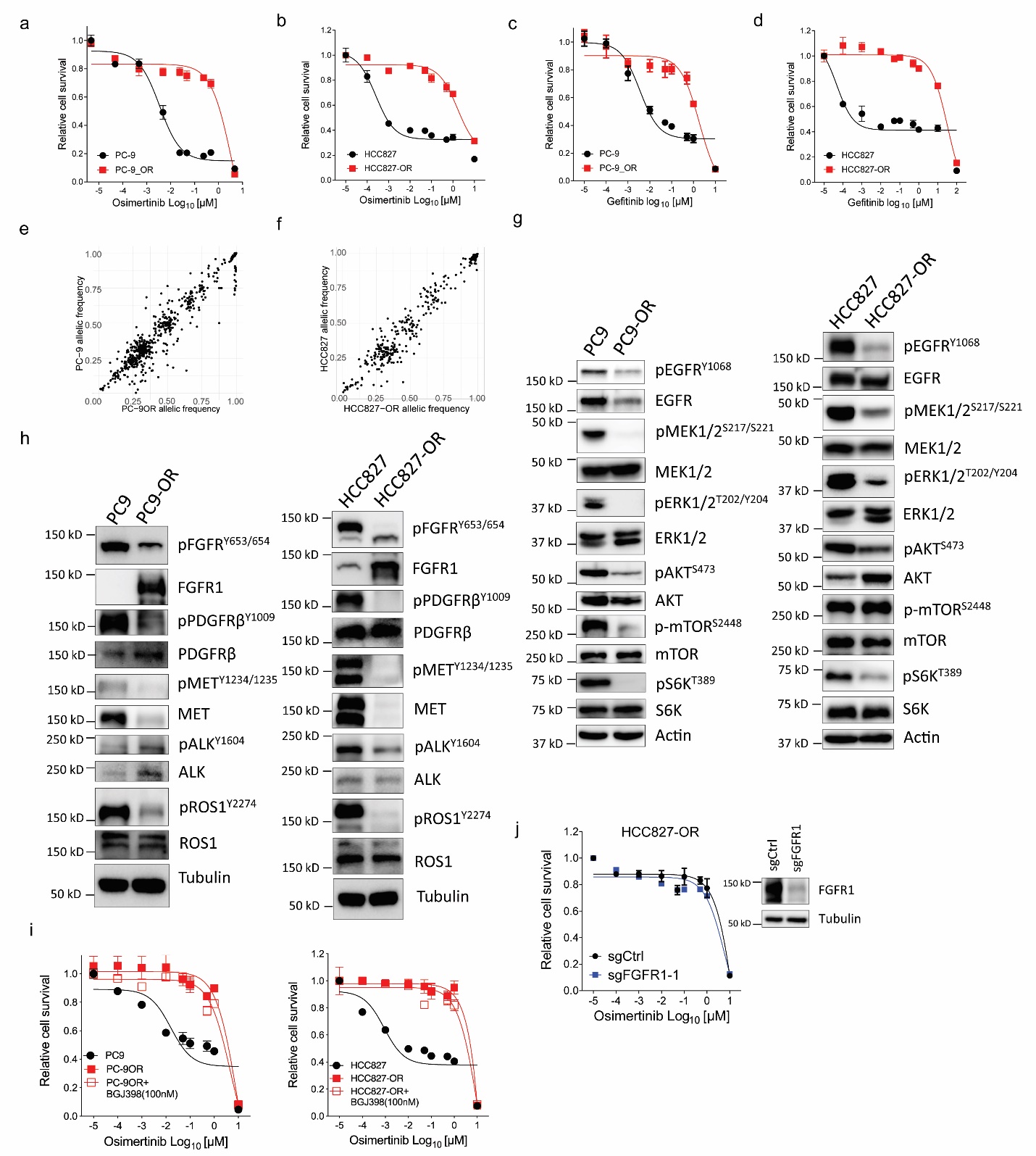


**Figure S1. Characteristics of *EGFR^mut^* lung cancer cells resistant to osimertinib.** **a**, **b**, Relative survival curve describing the viability of PC-9 and PC-9OR (**a**) or HCC827 and HCC827-OR cell lines (**b**) treated with the indicated concentrations of the osimertinib for 72 h. **c**, **d**, Relative survival curve describing the viability of PC-9 and PC-9OR (**c**) or HCC827 and HCC827-OR cell lines (**d**) treated with the indicated concentrations of the gefitinib for 72 h. **e**, **f**, Two-dimensional density plot of single-nucleotide variant allelic frequencies from whole exome sequencing (WES) of osimertinib resistant versus treatment-naive PC-9 (**e**) and HCC827 (**f**) cells. **g**, **h**, IB analyses to determine the activity of EGFR and its downstream pathway (**g**) and other TKI-resistant related RTKs (**h**) in treatment naïve and osimertinib resistant cell lines. **i**, Relative survival curve describing the viability of PC-9OR (left panel) and HCC827-OR (right panel) cells with or without the presence of FGFR inhibitor (BGJ398, 100nM) treated with the indicated concentrations of the osimertinib for 72 h. **j**, Relative survival curve describing the viability of HCC827-OR with or without the infection of sgFGFR1 lentivirus treated with the indicated concentrations of the osimertinib for 72 h (left panel). Immunoblots showed the knockout efficiency of sgFGFR1 lentivirus in HCC827-OR cells (right panel).


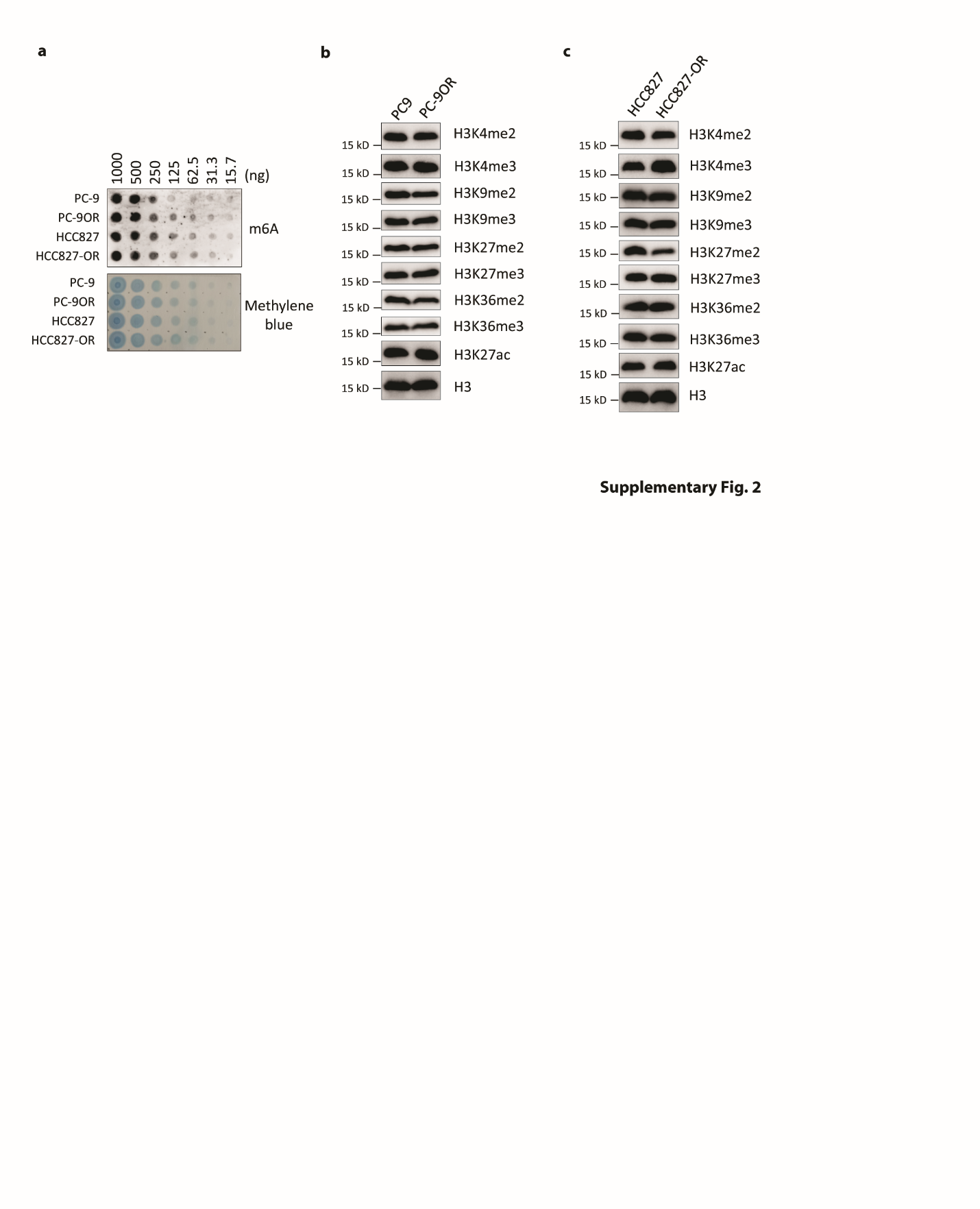


**Figure S2. Modifications on m^6^A and histone have no differences between osimertinib-resistant cells and their parental counterparts.** **a**, Global m^6^A levels in osimertinib-resistant (OR) and its paired treatment naïve PC-9 and HCC827 cell lines were determined by dot blot assay. The methylene blue staining was used as total RNA loading control. **b**, **c**, Treatment naïve and its paired OR cell lines of PC-9 (**b**) and HCC827 (**c**) was subjected to immunoblot (IB) analysis.


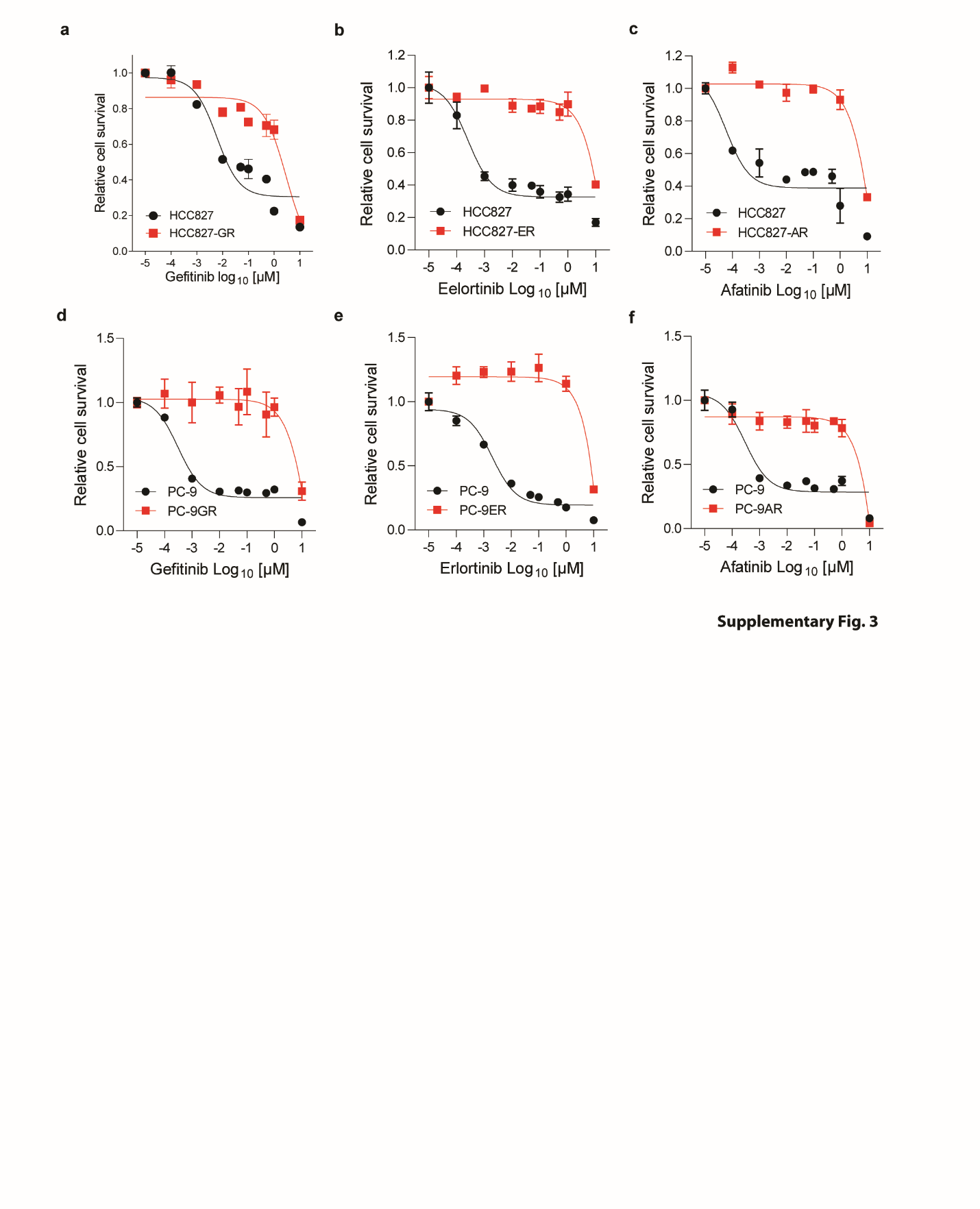


**Figure S3. Cell viability assay to confirm the EGFR-TKI resistance in *EGFR^mut^* lung cancer cells.** **a**-**f**, Relative survival curve describing the viability of HCC827 and HCC827-GR (**a**), or HCC827-ER (**b**), or HCC827-AR (**c**); PC-9 and PC-9GR (**d**), or PC-9ER (**e**), or PC9-AR (**f**) cell lines treated with gradient-increasing concentrations of the indicated EGFR-TKI for 72 h. GR, gefitinib-resistant. ER, erlotinib-resistant. AR, afatinib-resistant. OR, osimertinib resistant.


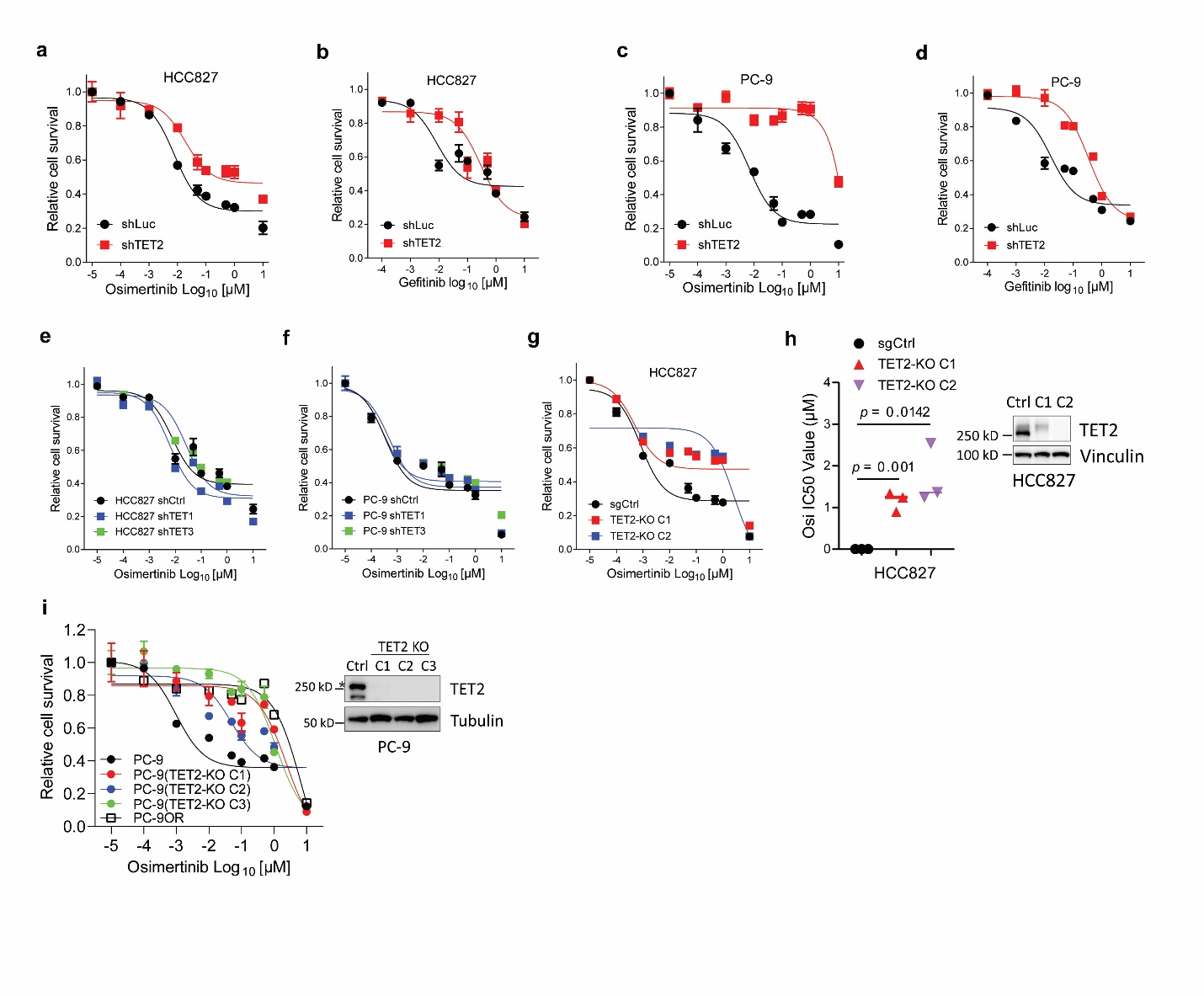


**Figure S4. TET2 deficiency elicits drug resistance to EGFR-TKIs in *EGFR^mut^* lung cancer cells. a**, **b**, Relative survival curve describing the viability of HCC827 cells with or without the knockdown of *TET2* by shRNA treated with the indicated concentrations of the osimertinib (**a**) or gefitinib (**b**) for 72 h. **c**, **d**, Relative survival curve describing the viability of PC-9 cells with or without the knockdown of *TET2* by shRNA treated with the indicated concentrations of the osimertinib (**c**) or gefitinib (**d**) for 72 h. **e**, **f**, Relative survival curve describing the viability of HCC827 (**e**) or PC-9 (**f**) cells with or without the knockdown of *TET1* or *TET3* by shRNA treated with the indicated concentrations of the osimertinib for 72 h. **g**, Relative survival curve describing the viability of two *TET2* knockout HCC827 clones and the parental cells transduced with sgCtrl lentivirus treated with the indicated concentrations of the osimertinib for 72 h. **h**, IC50 values of osimertinib in indicated cells as described in **g**. Right panel, immunoblots to confirm the knockout efficiency of *TET2* in HCC827 cells. **i**, Relative survival curve describing the viability of three *TET2* knockout PC-9 clones and the parental cells transduced with sgCtrl lentivirus treated with the indicated concentrations of the osimertinib for 72 h. Right panel, immunoblots for confirming the knockout efficiency of *TET2* in PC-9 cells. The asterisk indicates the main band of TET2. *P* values were calculated using two-tailed unpaired Student’s *t*-tests (**h**).


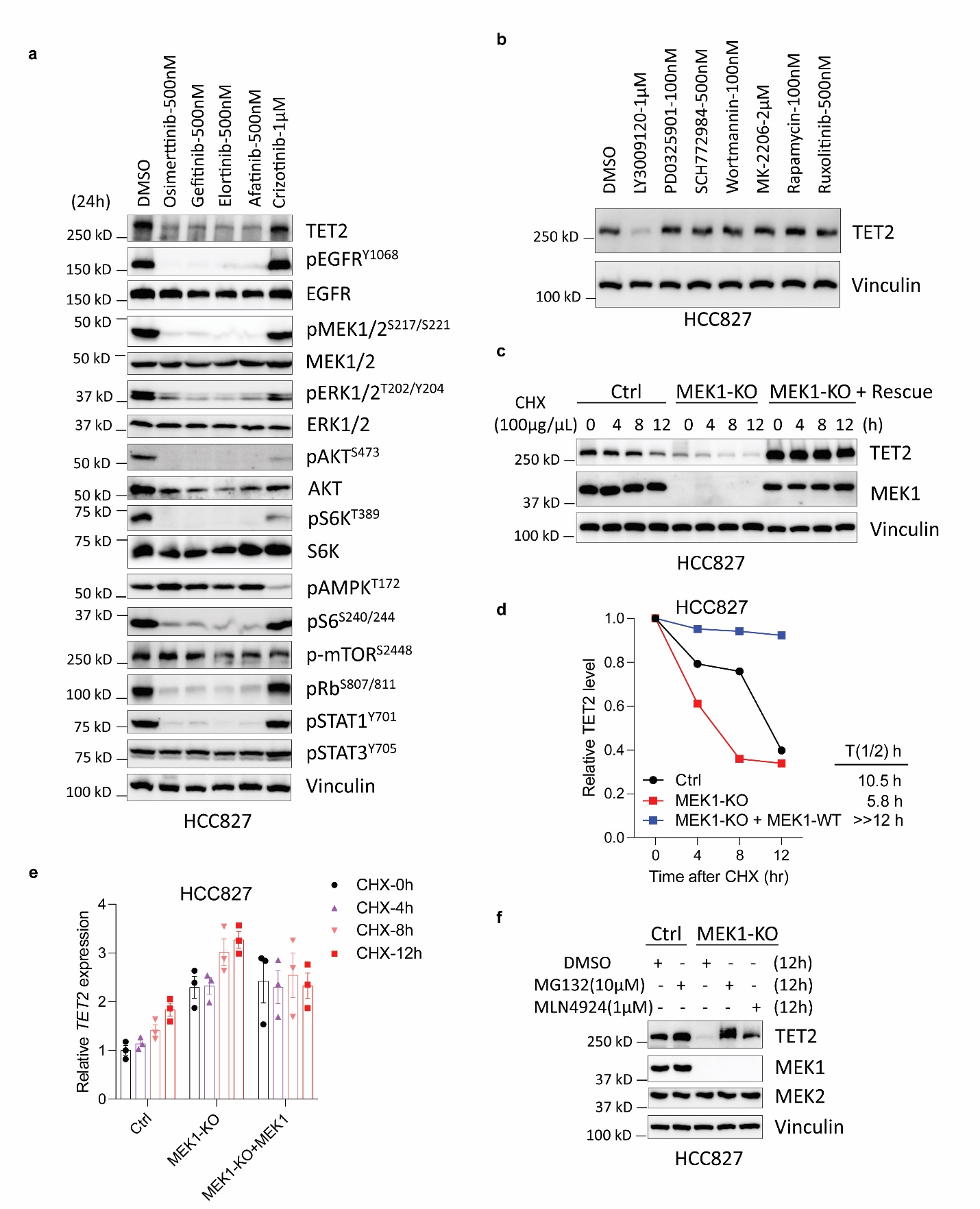


**Figure S5. MEK1 stabilize TET2 in a transcription independent manner. a**, Immunoblots to determine the TET2 level and EGFR-related downstream pathway in HCC827 cells treated with different TKIs. **b**, Immunoblots to determine the TET2 level in HCC827 cells treated with a series of inhibitors targeting BRAF (LY3009120), MEK (PD0325901), ERK (SCH772984), PI3K (Wortmannin), Akt (MK-2206), mTOR (Rapamycin), JAK-STAT (Ruxolitinib). **c**, HCC827 or HCC827^MEK1-KO^ cells with or without MEK1 rescue were treated with 100 μg/mL CHX (cyclohexane) prior to IB analysis. Vinculin served as a loading control. **d**, TET2 protein levels as described in **c** were quantified by normalization to vinculin. Time of TET2 half-life (T1/2) in each group was denoted. **e**, Relative *TET2* mRNA level in indicated cells as described in **c**. **f**, HCC827 or HCC827^MEK1-KO^ cells with or without the treatment by MG132 (10μM) or MLN4924 (1μM) for 12h prior to IB analysis.


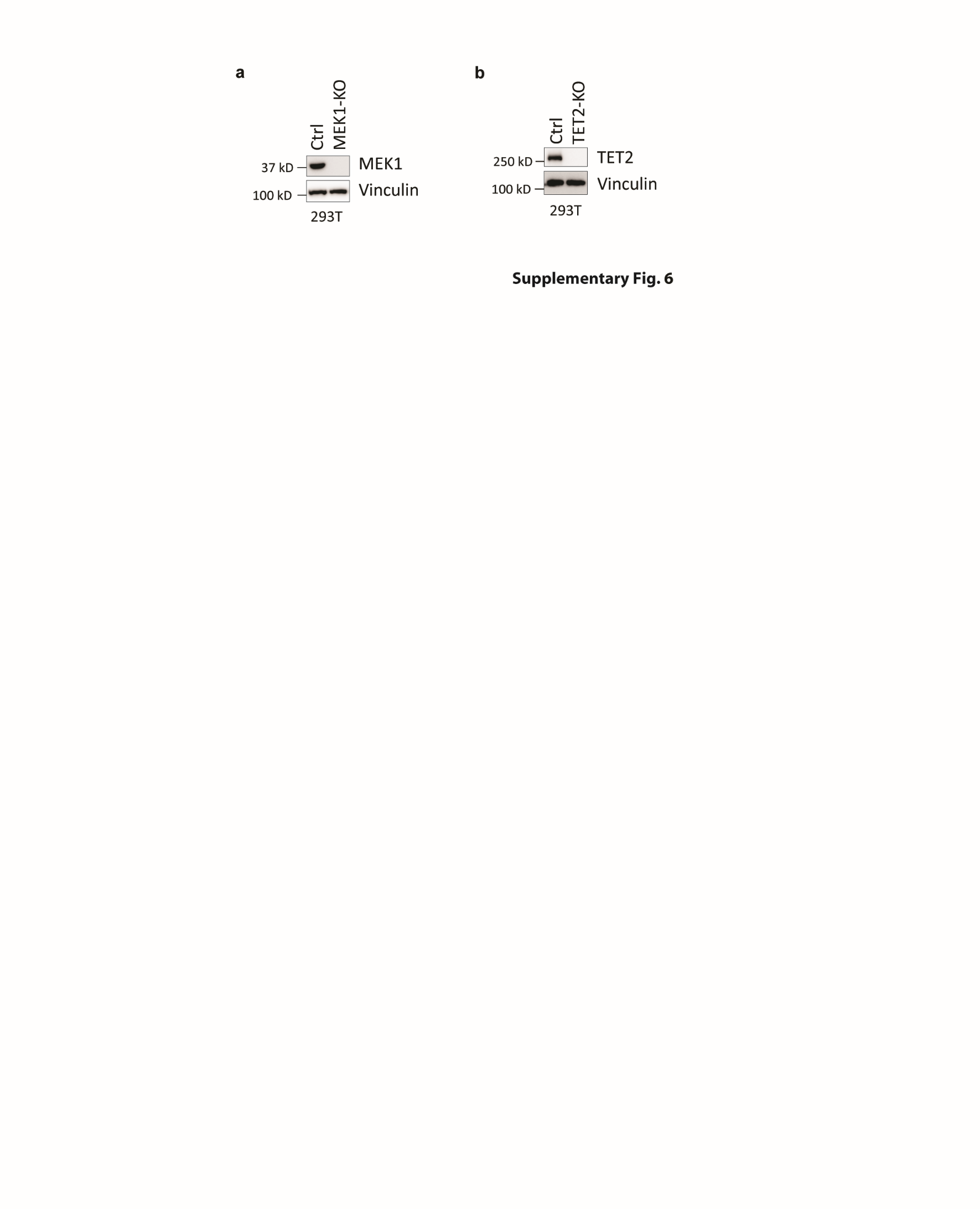


**Figure S6. Knockout of *MEK1* or *TET2* gene in 293T cells.** **a**, Immunoblots to measure the knockout efficiency of *MEK1* in 293T cells. **b**, Immunoblots to measure the knockout efficiency of *TET2* in 293T cells. Vinculin served as a loading control.


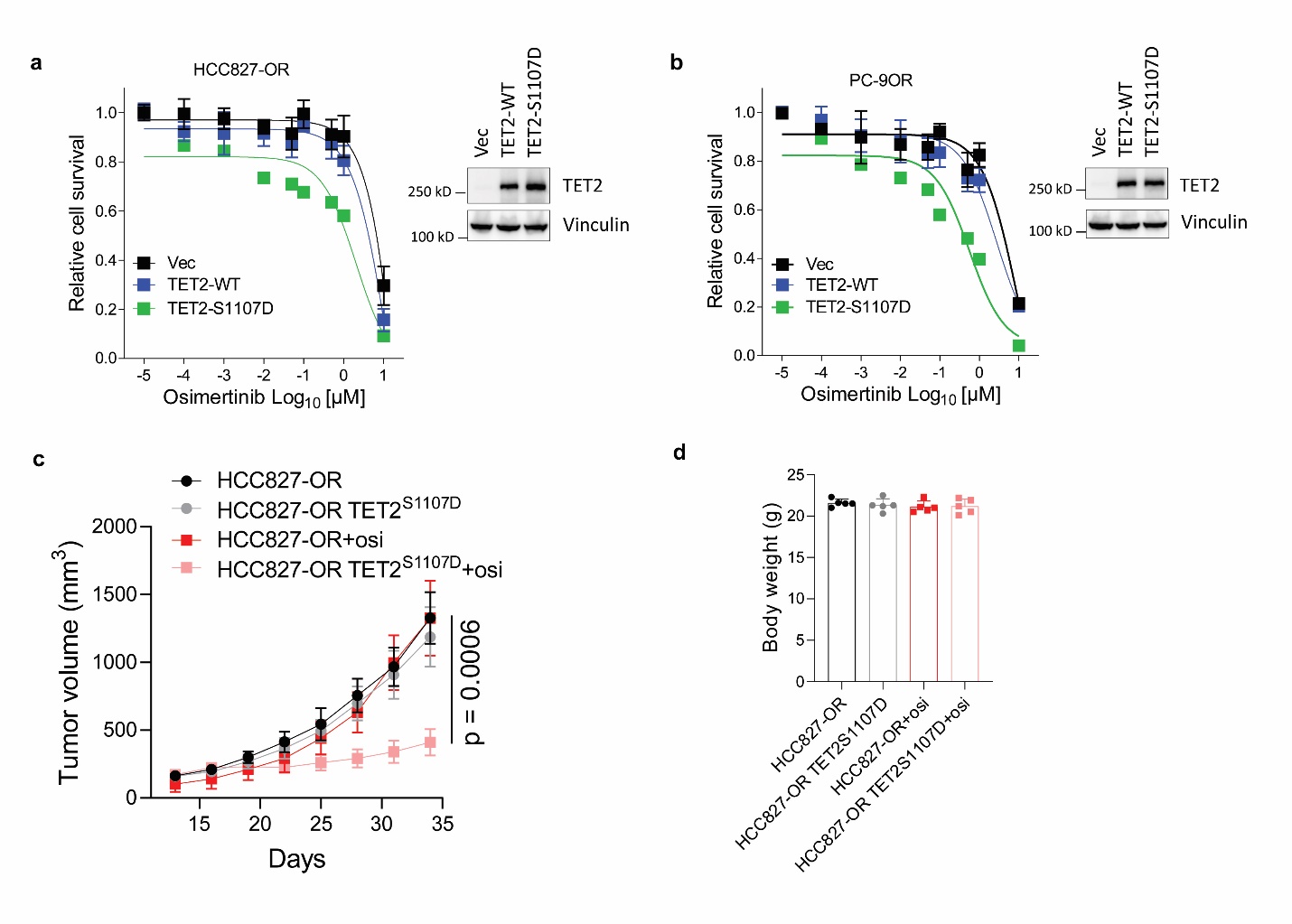


**Figure S7. TET2^S1107D^ but not TET2^WT^ could partially rescue the sensitivity to osimertinib in resistant cells. a**, **b**, Relative survival curve describing the viability of HCC827-OR (**a**) or PC-9OR (**b**) cells without or with the overexpression of TET2^WT^ or TET2^S1107D^ treated with the indicated concentrations of the osimertinib for 72 h. **c**, Growth curves of xenograft tumors derived from HCC827-OR cells or HCC827-OR introduced with TET2^S1107^ and then treated with or without osimertinib (2.5 mg/kg, i.p., daily). **d**, Body weight of mice described in **c** at the endpoint of treatment.

**
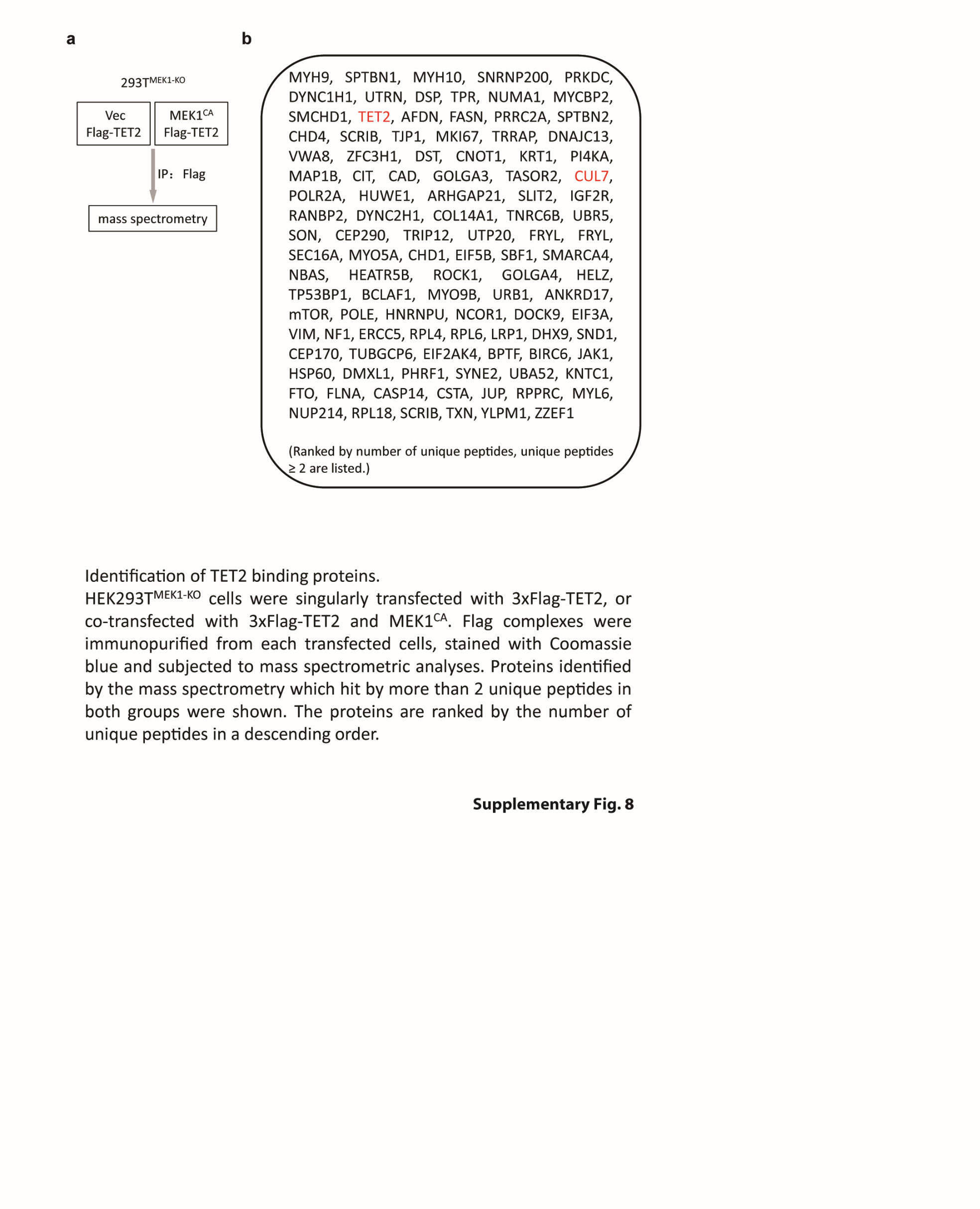
**

**Figure S8. Identification of the proteins interacted with TET2.** **a**, Flow chart to show Flag IP products from 293T^MEK1-KO^ cells transfected with indicated constructs and treated with MG132 were subjected to mass spectrometry. **b**, Proteins identified by the mass spectrometry which were hit by more than 2 unique peptides in both groups were shown. The proteins were ranked by the number of unique peptides in a descending order.

**
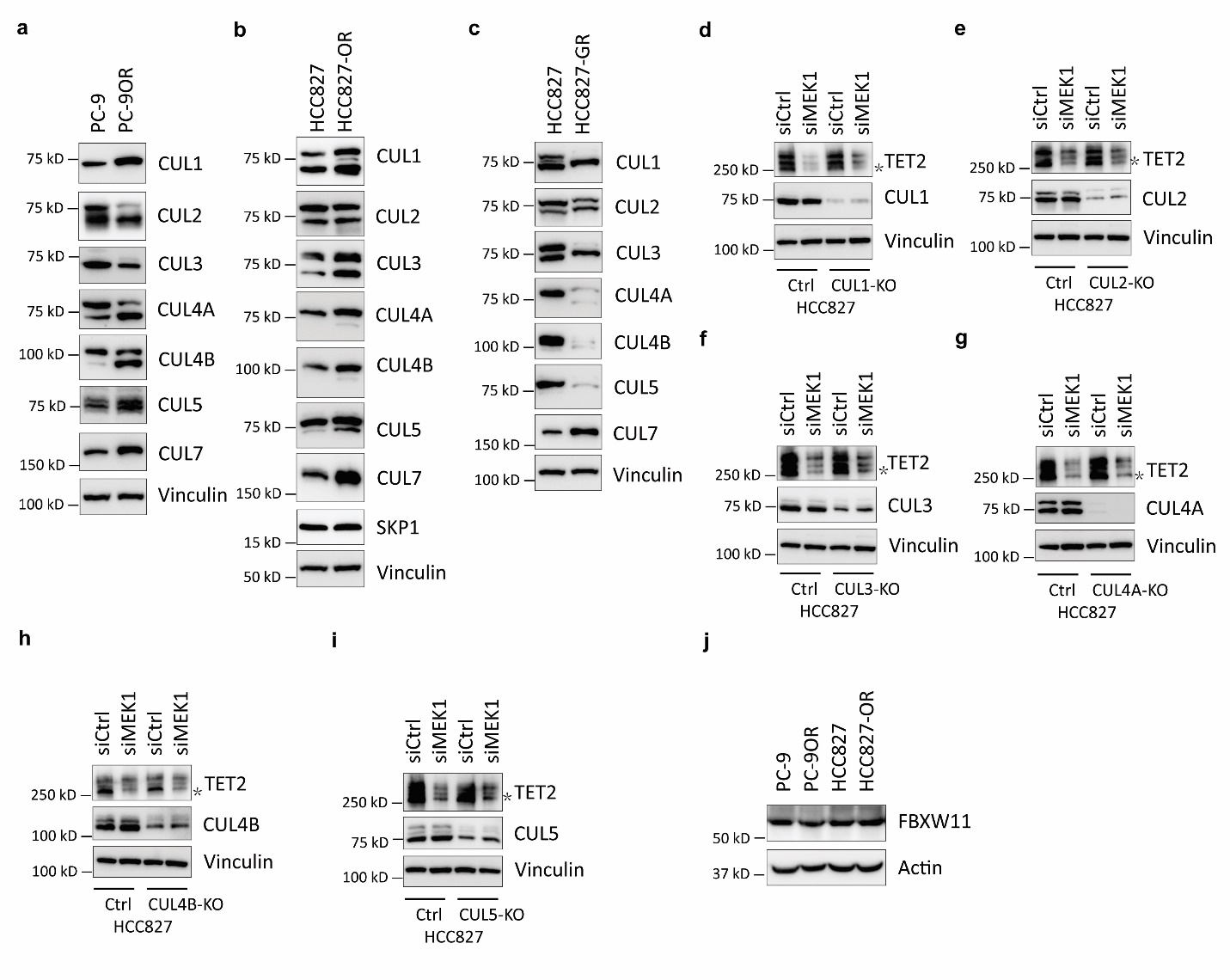
Figure S9. Screening of cullins that are associated with TET2 proteasome degradation.** **a**-**c**, Immunoblots of cullins in PC-9OR (**a**), HCC827-OR (**b**), HCC827-GR (**c**) and the corresponding parental cells. **d**-**i**, IB analyses to determine the TET2 level in HCC827 cells without or with the knockout of *CUL1* (**d**), or *CUL2* (**e**), or *CUL3* (**f**), or *CUL4A* (**g**), or *CUL4B* (**h**), or *CUL5* (**i**) gene treated with indicated siRNAs. The asterisks indicate the main band of TET2, which was slightly higher than the position of 250 kD. The bands with higher position were unspecified, which may represent other unknown post-translational modifications of TET2. **j**, Immunoblots to measure the FBXW11 level in indicated cells. For immunoblots of CUL1, CUL2, CUL3, CUL4A, CUL4B and CUL5, two or more bands were observed. According the product manual, the lowest band corresponds to the protein itself while the higher band(s) represent the neddylated form.

**
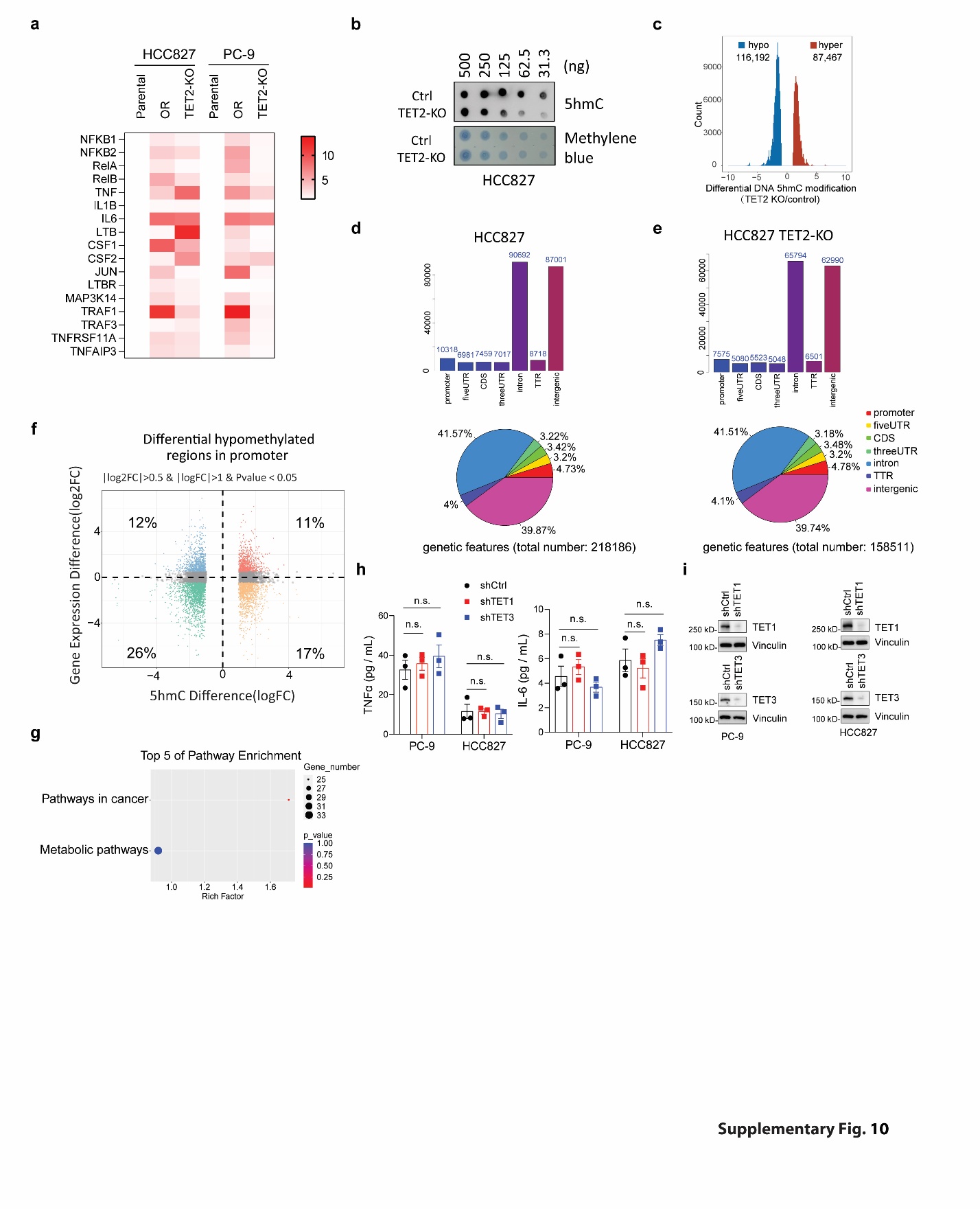
Figure S10. Loss of *TET2* resulted in decreased global level of 5hmC and up-regulated the TNF/NF-κB pathway.** **a**, Heatmap to show the differential gene expression of NF-κB pathway in indicated cell lines. The value displayed in the right symbols represents the fold change relative to control. **b**, Global 5hmC levels in HCC827^TET2-KO^ and HCC827 cell were determined by dot blot assay. The methylene blue staining was used as total genomic DNA loading control. **c**, Differential 5hmC modifications in HCC827 cells with TET2 KO. Hypo, hypo-hydroxymethylated sites; Hyper, hyper-hydroxymethylated sites. **d**, **e**, Distribution of hypomethylated regions in HCC827 (**d**) and HCC827^TET2-KO^ (**e**) cells among various genomic elements. Upper, histogram. Bottom, pie chart. **f**, Scatter plots to show the differential gene expression and the differential hypomethylated regions in promoter derived from integrated analysis of RNA-Seq and 5hmC-Seq data in HCC827 and HCC827^TET2-KO^ cells. **g**, KEGG analyses of genes within differential hypomethylated regions in promoter in HCC827^TET2-KO^ cells, the gene sets are shown as blue in **f**. **h**, ELISA to measure TNFα and IL-6 level in PC-9 and HCC827 with the RNA interference of *TET1* or *TET3*. **i**, Immunoblots to measure the knockdown efficiency of TET1 and TET3 in indicated cells.

**
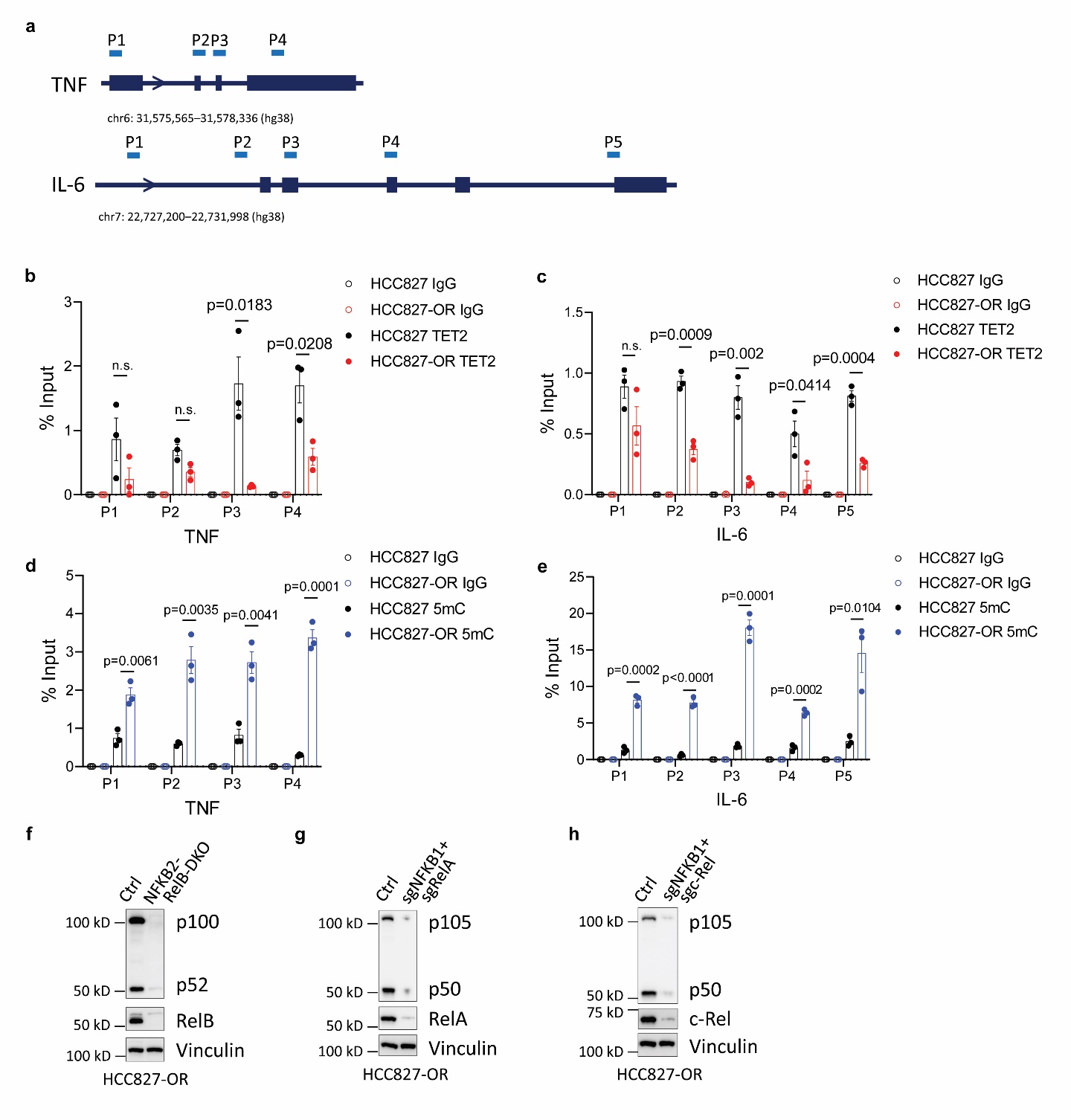
Figure S11. Loss of *TET2* up-regulated TNF/****NF-κB pathway through impaired DNA demethylation. a**, Illustration of *TNF* and *IL-6* gene. Primers designed for ChIP-qPCR are denoted. **b**, **c**, ChIP-qPCR of the TET2 enrichment on *TNF* (**b**) or *IL-6* (**c**) gene regions relative to immunoglobulin G (IgG) in indicated cells. **d**, **e**, ChIP-qPCR of the 5mC enrichment on *TNF* (**d**) or *IL-6* (**e**) gene regions relative to IgG in indicated cells. **f**-**h**, Immunoblots to show the knockout efficiency of key factors in NF-κB pathway in HCC827-OR cells. n.s., no significant. *P* values were calculated using two-tailed unpaired Student’s *t*-tests (**b**-**e**).

**
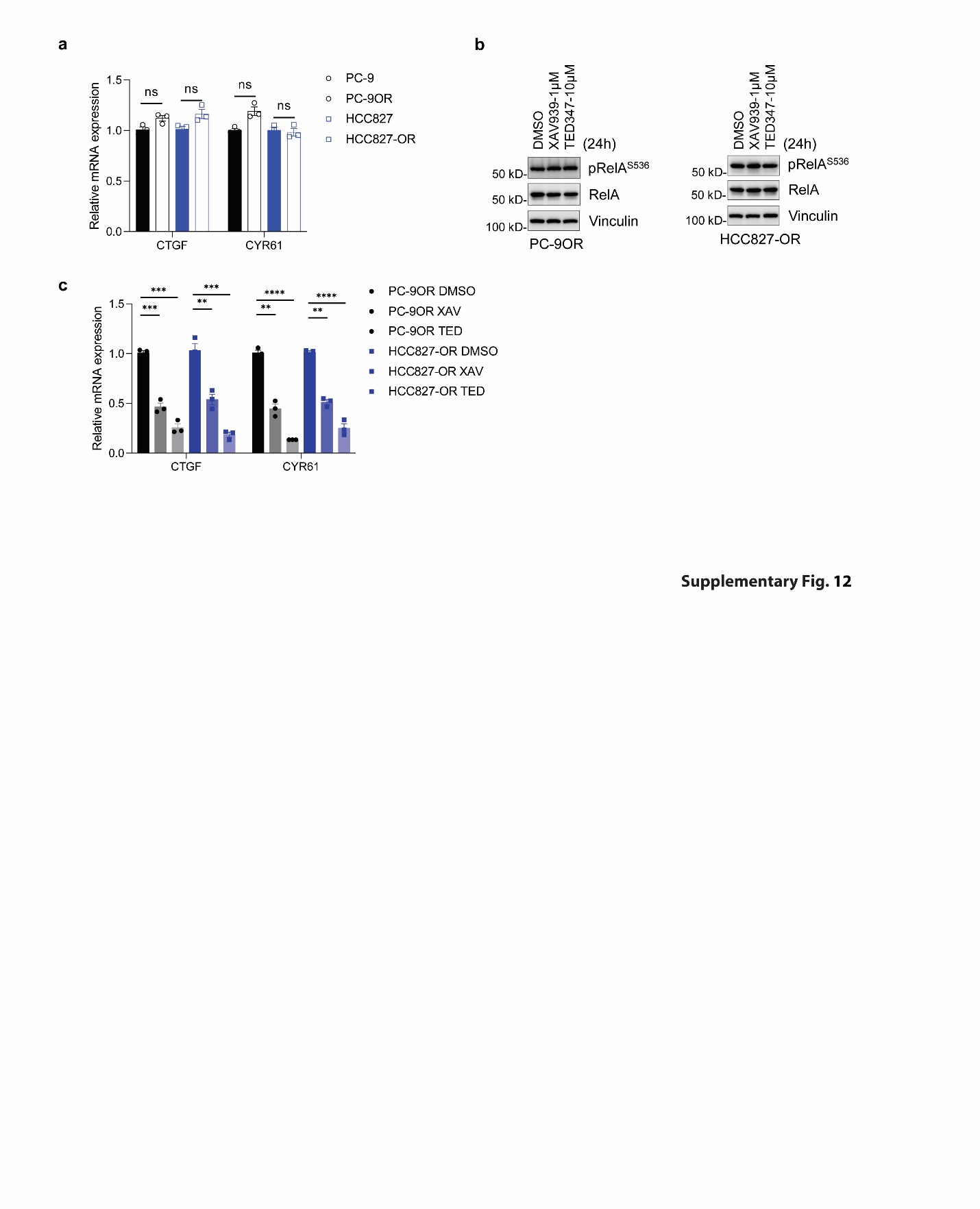
Figure S12. The NF-κB signaling in EGFR-TKI resistant cells was not affected by YAP/TEAD. a**, Relative mRNA level of *CTGF* and *CYR61* in indicated cells. **b**, Immunoblots of pRelA^S536^/RelA in PC-9OR and HCC827-OR cells treated with YAP/TEAD inhibitors (XAV939 or TED-347) for 24 hrs. **c**, Relative mRNA level of CTGF and CYR61 in cells described in **b**.
