# Supplementary figures and images for: Tet methylcytosine dioxygenase 2 (TET2) deficiency elicits EGFR-TKI (tyrosine kinase inhibitors) resistance in non-small cell lung cancer
Source: Signal Transduct Target Ther. 2024 Mar 9;9:65. doi: 10.1038/s41392-024-01778-4 (PMC10924974; doi:10.1038/s41392-024-01778-4)

Fig.3a

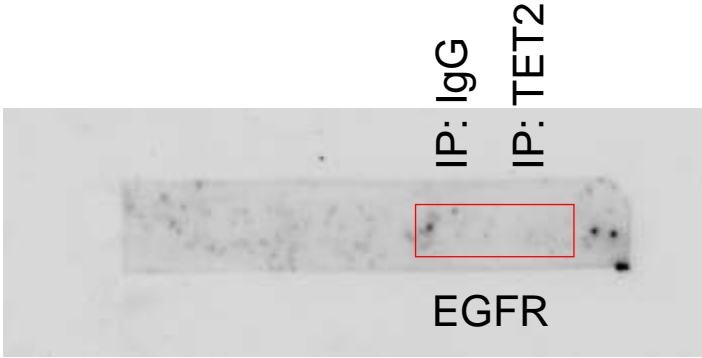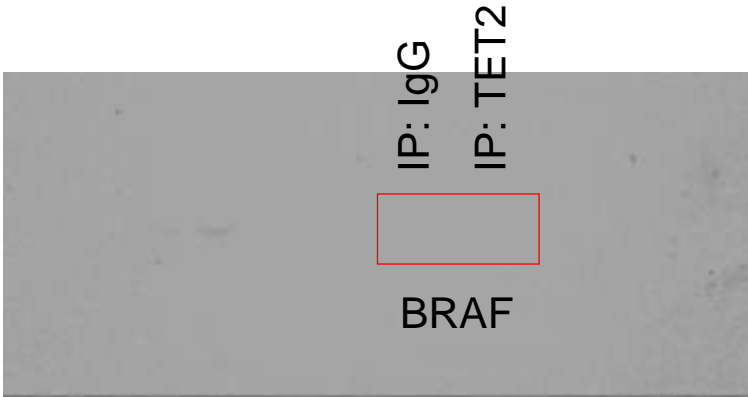

Supplement: Supplementary file 2 — Part of unprocessed WB images [file 41392_2024_1778_MOESM2_ESM.pdf]
